# Recent Advances in Field‐Effect Transistor‐Based Biosensors for Label‐Free Detection of SARS‐CoV‐2

**DOI:** 10.1002/smsc.202300058

**Published:** 2023-12-21

**Authors:** Haiyang Yu, Huibin Zhang, Zhe Liu, Linrun Feng, Yuezeng Su, Jinhua Li, Wei Tang, Feng Yan

**Affiliations:** ^1^ Department of Electronic Engineering Shanghai Jiao Tong University Shanghai 200240 China; ^2^ Hangzhou LinkZill Technology Co., Ltd. Hangzhou Zhejiang 311121 China; ^3^ Collaborative Innovation Center for Advanced Organic Chemical Materials Co-constructed by the Province and Ministry Key Laboratory for the Green Preparation and Application of Functional Materials Ministry of Education Hubei Key Laboratory of Polymer Materials, School of Materials Science and Engineering Hubei University Wuhan 430062 China; ^4^ Department of Applied Physics and Research Institute of Intelligent Wearable Systems The Hong Kong Polytechnic University Kowloon Hong Kong 999077 China

**Keywords:** biosensors, electrochemical detection, field-effect transistors, label-free, SARS-CoV-2

## Abstract

Field‐effect transistor‐based biosensors (FET biosensors) have gained significant attention in the context of the global SARS‐CoV‐2 pandemic owing to their label free and highly sensitive detection capabilities. In this comprehensive review, recent advances in the use of electrochemical‐transistor‐based biosensors for the label‐free detection of SARS‐CoV‐2 are analyzed. A general introduction to the electrochemical‐transistor‐based biosensor system is initiated, highlighting its critical technical requirements. Various structural designs and working principles of transistor‐based transducers are described, and the essential aspects of interface engineering for improved sensing performance are summarized. The applications of transistor‐based biosensors for the detection of SARS‐CoV‐2 are discussed in detail. Finally, perspectives for the future development of transistor‐based sensing systems are provided. This review aims to provide valuable insights and guidance for the design and optimization of biochemical sensor systems with the potential to impact health monitoring, disease diagnosis, and biosafety in the field of in vitro diagnostic products.

## Graphical abstract

Recent advances in the use of field‐effect transistor (FET)‐based biosensors for label‐free detection of SARS‐CoV‐2 are analyzed. The system's technical requirements, transducer designs, and interface engineering are covered, various applications are demonstrated, and insights are provided into the future development of FET sensing systems. The review aims to guide the design of biochemical sensor systems and their potential impact on healthcare.

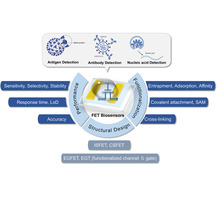

## 1 Introduction

COVID‐19 is caused by the single‐stranded RNA virus, SARS‐CoV‐2.^[^ ^1^, ^2^ ^]^ By February 2020, a global coronavirus pandemic had begun to unfold.^[^ ^3^ ^]^ Currently, the Omicron variant (lineage B.1.1.529), which has been designated as a variant of concern (VOC) by the World Health Organization (WHO), has rapidly become the dominant variant worldwide.^[^ ^4^ ^]^ Because of the continuing evolution of SARS‐CoV‐2, the emergence of new variants is not a rare event but occurs periodically.^[^ ^5^ ^]^ The spread of the virus has resulted in significant morbidity and mortality globally.^[^ ^6^ ^]^ CoVs have some of the largest genomes among the RNA viruses, ranging from 26 to 32 kb. Upon cell entry, the viral RNA is translated to produce nonstructural proteins (NSPs) from two open‐reading frames (ORFs): ORF1a and ORF1b.^[^ ^7^ ^]^ The SARS‐CoV‐2 genome encodes 29 known proteins, including four structural proteins: spike (S), membrane (M), envelope (E), and nucleocapsid (N). Each S protomer is composed of the S1 and S2 subunits and a single transmembrane (TM) anchor. The S protein binds to the cell surface receptor angiotensin‐converting enzyme 2 (ACE2) through the receptor‐binding domain (RBD), which is a crucial step in membrane fusion.^[^ ^8^ ^]^ During the initial days of the pandemic, lung infection was considered one of the main symptoms of SARS‐CoV‐2 infection. Chest computed tomography (CT) was used to diagnose COVID‐19 by identifying ground‐glass opacities.^[^ ^1^ ^]^ However, later it was found that SARS‐CoV‐2 was more likely to infect the upper respiratory tract than the distal lungs.^[^ ^9^ ^]^ Omicron replicates faster than all the other studied SARS‐CoV‐2 variants in the bronchi but less efficiently in the lung parenchyma,^[^ ^10^ ^]^ resulting in reduced lung infectivity and pathogenicity.^[^ ^11^ ^]^ Therefore, the diagnosis of lung infection does not meet the requirements for the rapid screening of potentially infected individuals. Reverse transcription polymerase chain reaction (RT‐PCR) is currently the gold standard for COVID‐19 diagnosis.^[^ ^12^ ^]^ Approved nucleic acid testing products target ORF1ab, envelope protein (E), and nucleocapsid protein (N) regions of the viral genome. Although different products have the same detection principle, their primer and probe designs vary, and they differ in the detection and interpretation of single (ORF1ab), dual (ORF1ab, N, or E), and triple targets (ORF1ab, N, and E). Coronavirus spike (S) glycoproteins facilitate the entry of the virus into cells and are the primary targets of antibodies.^[^ ^13^ ^]^ Rapid antigen test (RAT) and rapid antigen self‐testing (RAST) are easy‐to‐use diagnostic tools for detecting high viral loads.^[^ ^14^, ^15^ ^]^ Lateral flow tests (LFTs) are typically used to detect viral protein (antigen) of SARS‐CoV‐2. The use of LFTs for RAST has increased worldwide.^[^ ^16^ ^]^ However, the lowest amount of virus detectable using LFTs and RT‐PCR can differ by several orders of magnitude.^[^ ^15^ ^]^ Antibody‐based methods targeting IgG and IgM induced by the recombinant N and S proteins of SARS‐CoV‐2 correlate with the results of nucleic acid‐based assays. Additionally, a recent study demonstrated that the serum IgA level in COVID‐19 patients was positively correlated with disease severity,^[^ ^17^ ^]^ indicating that serum IgA can be used as a biological marker for COVID‐19 identification.

In recent years, a surge has occurred in the development of SARS‐CoV‐2 detection methods based on various molecular recognition techniques such as enzyme‐linked immunosorbent assays (ELISAs),^[^ ^18^, ^19^, ^20^ ^]^ lateral flow assays (LFAs),^[^ ^21^, ^22^, ^23^, ^24^ ^]^ electrochemical,^[^ ^25^, ^26^, ^27^ ^]^ and optical technologies.^[^ ^28^, ^29^, ^30^ ^]^ Among these techniques, transistor‐based biosensors have shown rapid progress as point‐of‐care (POC) tools owing to their high sensitivity, fast analysis, compatibility with microfabrication techniques, low power consumption, and scalability. Transistors offer several advantages for biodetection: 1) a relatively low operating voltage that prevents unnecessary redox and water cleavage reactions, making them suitable for analyzing biological samples in solution; 2) transistor‐based biosensors are highly sensitive owing to their inherent signal amplification; and 3) they can be miniaturized and integrated into high‐density arrays for multichannel high‐throughput analyses and as wearable sensing devices.^[^ ^31^ ^]^ Transistors have been used in various platforms for detecting biological substances such as nucleic acids,^[^ ^32^, ^33^, ^34^ ^]^ proteins,^[^ ^35^, ^36^ ^]^ small molecules,^[^ ^37^, ^38^ ^]^ virus particles,^[^ ^39^, ^40^ ^]^ cells,^[^ ^41^, ^42^, ^43^ ^]^ and others. The COVID‐19 pandemic has highlighted the importance of disease prevention and control, with the timely detection of pathogens or related biomarkers playing a crucial role. Transistor‐based biosensors are emerging detection platforms that are receiving continuous attention and research with rapid developments in both depth and breadth. There is a growing trend toward engineering transistor‐based biosensors that are integrated as crucial sensing elements in various biosensor systems, catering to the diverse needs of different equipment.^[^ ^44^ ^]^ Integration of cutting‐edge technologies such as the Internet of Things and artificial intelligence has enabled the collection of vast amounts of analyzable data that can be utilized to diagnose and prevent numerous diseases, thereby revolutionizing the healthcare industry.^[^ ^45^, ^46^ ^]^

This review provides a thorough analysis of the use of transistor‐based biosensors for the detection of SARS‐CoV‐2. First, a general introduction to the electrochemical transistor‐based biosensor system is presented, outlining its critical technical requirements. Various structural designs and working principles of transistor‐based transducers have been described. Subsequently, the functionalization of various sensitive substrates is summarized to demonstrate the importance of interface engineering for the improvement of sensing performance. Subsequently, the applications of transistor‐based biosensors for the detection of SARS‐CoV‐2 are discussed in detail. Finally, the perspectives on the future development of transistor‐based sensing systems are discussed.

## 2 Electrochemical Transistor Biosensor Systems

### 2.1 Biosensor System Components

**Figure 1:**
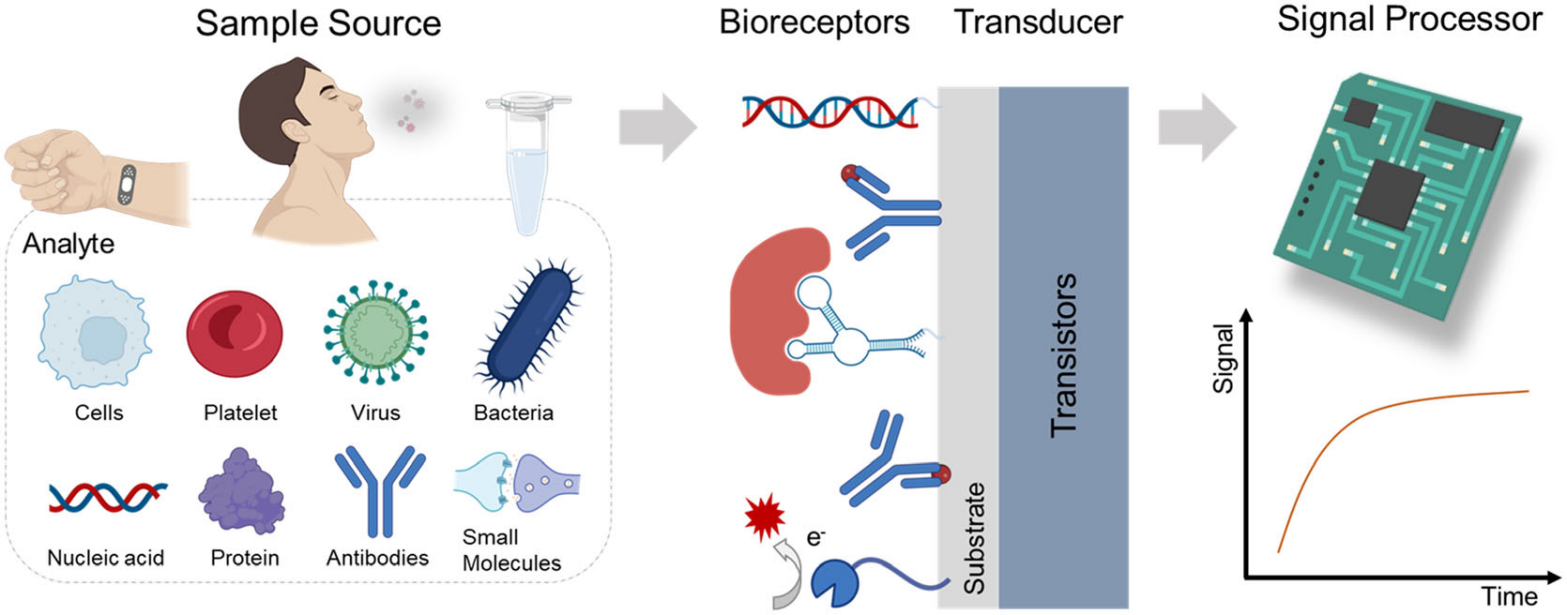
A schematic of a transistor‐based electrochemical biosensor system.

Biosensor devices consist of bioactive materials as receptors and transducers capable of generating chemical, optical, or electrical signals in response to biological stimuli. Advances in multidisciplinary fields such as chemistry, physics, biology, and materials science have led to the emergence of various biosensing tools with diverse analytical properties, among which electrochemical biosensors are regarded as promising detection techniques. A typical electrochemical biosensor system comprises three main components: a recognition element, transducer, and signal processor, as shown in **Figure** **1**.^[^ ^47^, ^48^ ^]^ Different samples, such as saliva, aerosols, serum, and nasopharyngeal swabs, are used with different target molecules that can be detected directly or need to be extracted and purified.^[^ ^26^, ^39^, ^49^, ^50^ ^]^ The recognition element of an electrochemical biosensor system includes stable biologically active receptors that interact with target analytes to produce corresponding changes in interfacial electrical performance. The transducer converts biochemical signal changes into measurable signals, which are further amplified, analyzed, and outputted by the circuit.^[^ ^51^, ^52^ ^]^ The concentration or content of the analyte measured using a biosensor may also be visually displayed in a portable system.^[^ ^53^ ^]^ Various bioactive materials such as antibodies,^[^ ^40^ ^]^ antigens,^[^ ^54^ ^]^ DNA,^[^ ^55^ ^]^ enzymes,^[^ ^56^ ^]^ and aptamers^[^ ^57^ ^]^ have been used as recognition elements for biosensors.

The development of micro‐and nanomanufacturing technologies has substantially contributed to the miniaturization and integration of microsensor systems based on diverse transistor transducers. Advances in nanotechnology and biotechnology have facilitated the development of efficient biosensors.^[^ ^58^, ^59^ ^]^ Field‐effect transistor‐based biosensors (FET biosensors) offer the advantages of high sensitivity, high specificity, low sample volume, rapid response, low cost, and label‐free detection.^[^ ^60^ ^]^ They are also capable of realizing integration applications in system‐on‐chip (SOC) technology, which allows the integration of recognition elements, transducers, signal processors, and other microcomponents on a single chip.^[^ ^61^ ^]^ Transistor‐based biosensing chips also enable the creation of lab‐on‐a‐chip (LOC) systems that automate the preparation, separation, reaction, and detection of biological samples, making them suitable for a wide range of applications such as field testing, online monitoring, and wireless sensor networks.^[^ ^62^ ^]^

### 2.2 Fundamental Metrics of Biosensors

**Figure 2:**
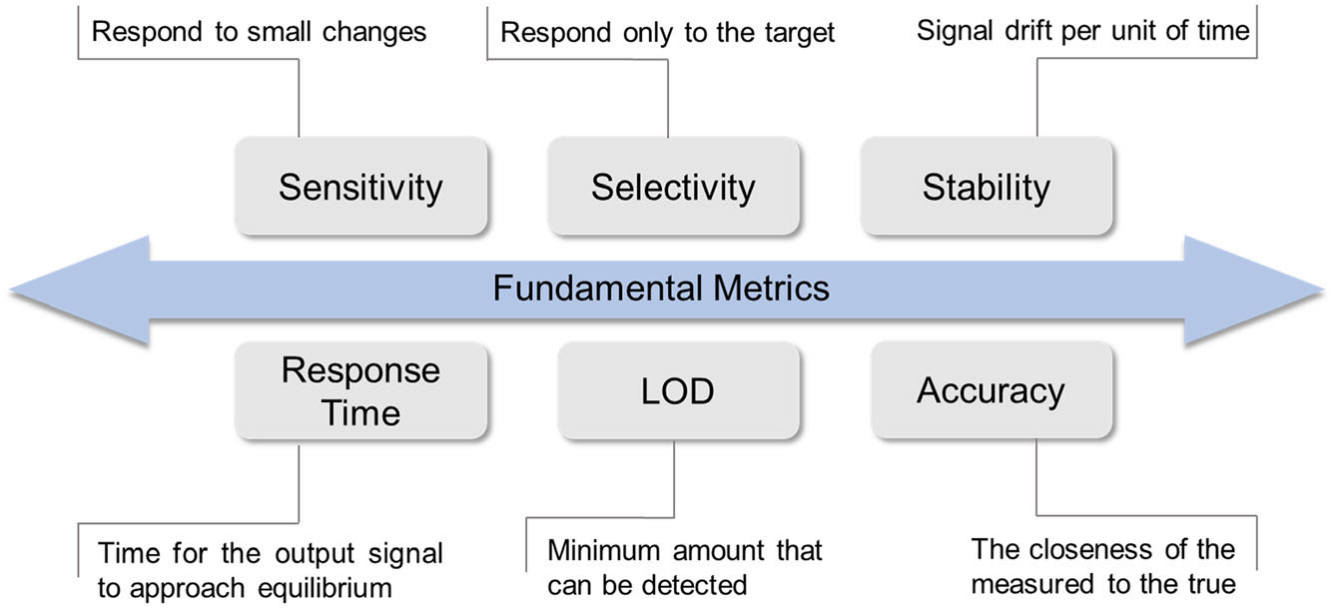
Overview of the fundamental metrics in the sensing performance of transistor‐based biosensors.

To evaluate the performance of biosensors, metrics such as sensitivity, selectivity, stability, response time, limit of detection (LoD), and accuracy are primary considerations (**Figure** **2**).^[^ ^48^, ^63^ ^]^

#### 2.2.1 Sensitivity

It reflects the ability of a biosensor to detect and respond to small changes in the analyte concentration, that is, the magnitude of the change in the sensor response signal corresponding to a change in the concentration or level of the target to be measured. The analytical sensitivity of the biosensor system is the slope of the calibration curve.^[^ ^64^ ^]^

#### 2.2.2 Selectivity

When testing for a specific analyte, the sample may contain a complex mixture of biological and chemical substances, such as proteins, ions, and microorganisms. Therefore, selectivity is of great importance, as it represents the ability of biosensors to respond only to the target analyte, despite the presence of potential competing analytes in the sample.^[^ ^65^, ^66^ ^]^ Selectivity is achieved through the use of a bioreceptor that specifically binds to the target analyte while minimizing nonspecific binding to other substances in the sample. Various techniques have been used to improve biosensor selectivity, including the use of suitable bioreceptors and optimization of surface chemistry and assay conditions.^[^ ^67^, ^68^ ^]^

#### 2.2.3 Stability

It ensures reproducible and reliable use of biosensors in changing environments, especially for long‐term monitoring. Therefore, it is essential to assess their stability over time with respect to the environmental factors.^[^ ^63^ ^]^ One way to measure stability is through signal drift, which is the degree of change in the output signal per unit of time while maintaining the same starting conditions.^[^ ^69^, ^70^ ^]^ The degree of variation between multiple output values reflects the stability of the biosensor.

#### 2.2.4 Response Time

It is defined as the time required for the sensor's electrical signal to reach 95% of its equilibrium value after adding the sample, which is an essential parameter for biosensor performance.^[^ ^48^ ^]^

#### 2.2.5 Limit of Detection

The LoD, which is another critical parameter, is the lowest concentration or value of a substance that can be detected using a sensor. The noise level, which is the signal generated by the sensor when analyzing a blank sample, is also a key factor to consider. For FET biosensors, the LoD is usually estimated as the concentration providing a response equal to two‐and‐a‐half or three times the noise level^[^ ^71^, ^72^ ^]^ or the noise level plus three times the noise standard deviation.^[^ ^73^, ^74^, ^75^ ^]^ In particular, the detection capability of the sensor needs to be estimated in a number of ways, from “yes/no” detection of the presence (LoD) to the minimal measurand amount that can be measured with defined accuracy (limit of quantification, LoQ).^[^ ^64^ ^]^

#### 2.2.6 Accuracy

The ability of a biosensor to quantitatively analyze a given test substance. The closeness of the measured values to the actual values reflects the accuracy of the sensor. The interplay between scientific inquiry and engineering optimization can enhance the sensor accuracy.^[^ ^63^ ^]^

Reproducibility (precision), linearity (signals that are directly proportional to the values of the measurand), and other metrics^[^ ^76^ ^]^ are often used for further performance evaluation. The precision of the biosensor describes the closeness of agreement between signals obtained using the same method but different fabricated biosensors, which are usually expressed numerically using the relative standard deviation (RSD).^[^ ^77^ ^]^

### 2.3 Requirement of Transistor Biosensors

The design of transistor‐based biosensors must satisfy several requirements to be effective in practical applications. First, the bioreceptor must be immobilized in a stable and reproducible manner to ensure accuracy and reliability of the sensing results. Second, the sensing surface must be biocompatible and exhibit high sensitivity and selectivity toward the target analyte. Third, the device must be capable of operating over a wide range of ion concentrations and temperatures without interference from the solution. Fourth, the device must be capable of detecting analytes in real or near real time for rapid and accurate analysis. Finally, the device must be cost effective and easy to integrate, making it suitable for mass production and commercialization.

## 3 Structural Design and Working Principle

FET biosensors have emerged as a promising platform for label‐free electrochemical biosensing applications. Generally, a transistor consists of a channel, a dielectric, and three terminals: source (S), drain (D), and gate (G) electrodes. The gate electrode was separated from the other two terminals by a thin insulator layer called the gate dielectric.^[^ ^78^ ^]^ A transistor‐based biosensor has a structure similar to that of a transistor, except that the bioreceptors are immobilized on a dielectric, electrode, or channel. The label‐free sensing mechanism of transistor‐based biosensors involves an interaction between the analyte and bioreceptor, resulting in changes in the surface potential. Because of the transducing capability of the transistor, the resulting alteration in the surface potential leads to a change in the current flow between the source and drain terminals. Label‐free detection eliminates the need for labeling or amplification, thereby reducing the complexity and cost of assays. Its fast response time and high sensitivity make it ideal for real‐time monitoring and analysis of biological and chemical substances.^[^ ^79^ ^]^ The low sample volume requirement for analysis makes it possible to detect analytes in small sample volumes. Its low manufacturing cost and ability to integrate with electronic circuits make it a promising tool in various fields, including biotechnology, medicine, environmental monitoring, food safety, healthcare, and biosecurity.

**Figure 3:**
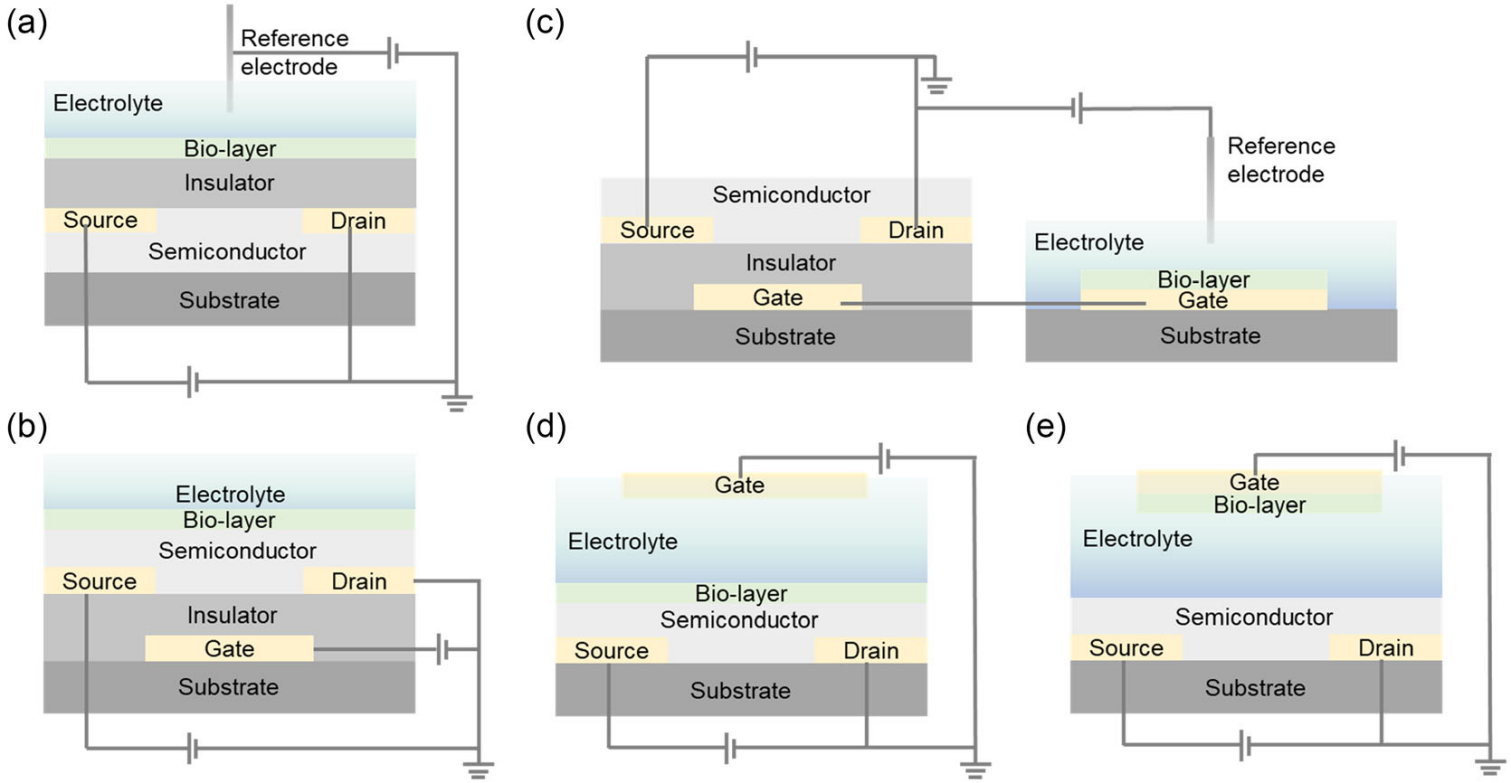
Different structural designs of transistor‐based biosensors. a) Conventional ion‐sensitive field‐effect transistor (ISFET) biosensor. b) Channel‐sensitive field‐effect transistor (CSFET) biosensor. c) Extended‐gate field‐effect transistor (EGFET) biosensor. d) Electrolyte‐gated transistor (EGT) biosensor with a functionalized channel. e) Electrolyte‐gated transistor (EGT) biosensor with a functionalized gate.

Transistor‐based biosensors can be categorized into five structural designs depending on the transistor type and immobilization location of the bioreceptor, as shown in **Figure** **3**. The ion‐sensitive field‐effect transistor (ISFET), which is the most conventional transistor biochemical sensor, is similar to the MOSFET; however, the metal gate is removed, leaving the insulator (SiO_2_) in direct contact with the solution (Figure 3a). ISFETs can be modified for biosensing by immobilizing bioreceptors on ion‐sensitive membranes, such as Si_3_N_4_, Al_2_O_3_, and Ta_2_O_5_.^[^ ^80^ ^]^ Various sensitive bioreceptors have been used to specifically detect a wide range of biomolecules.^[^ ^81^, ^82^, ^83^, ^84^, ^85^, ^86^, ^87^ ^]^ In addition to conventional ISFETs, organic field‐effect transistors (OFETs) can be implemented for ionic or biological sensitivity. Low‐cost organic semiconductor ISFETs have been proposed as replacements for Si‐based transistors. Ji et al.^[^ ^88^ ^]^ developed an ISFET based on the bottom‐contact architecture of a TFT using P3HT as the semiconductor and Ta_2_O_5_ as the dielectric for pH and potassium detection. However, because of the separation of the electrolyte from the active channel, ISFET‐based biosensors are sensitive only to the amount of charge accumulated at the semiconductor–dielectric interface.

A channel‐sensitive field‐effect transistor (CSFET) is another popular structure that exposes the channels of bottom‐gated transistors (Figure 3b). For such CSFET biosensors, it is possible to operate in a dry state or in an aqueous medium.^[^ ^89^, ^90^, ^91^ ^]^ The bioreceptor immobilization of CSFETs is implemented on the channel; thus, electrically coupled carriers in the channel upon receptor–analyte interaction usually have a higher impact on the total current, showing higher sensitivity than ISFET biosensors.^[^ ^92^ ^]^ However, degradation of the channel material, nonspecific adsorption, and solution ion interference are of great concern in the design of biosensors.^[^ ^93^, ^94^, ^95^ ^]^ To mitigate this challenge, an extended‐gate FET was employed by extending the sensing pad from the gate of a conventional FET device to the detection environment, as shown in Figure 3c. This structure allows multiple functionalization methods for the augmented gate without disrupting the device structure. However, this structure compromises the overall compactness of the device. For this configuration to work efficiently, the capacitance of the gate must be carefully considered, and the presence of parasitic capacitance may limit the sensitivity.^[^ ^96^ ^]^

Recently, biosensors have been constructed using electrolyte‐gated transistors (EGTs), which replace the gate capacitance dielectric with an electrolyte. In EGTs, the electrolyte is placed between the gate electrode and semiconductor channel, creating a double‐layer capacitance at the electrolyte/semiconductor interface. The use of an electrolyte as the gate dielectric allows for high‐capacitance and low‐gate‐voltage operation, which can lead to high sensitivity and low power consumption in biosensing applications.^[^ ^97^ ^]^ EGT functionalization can be implemented by immobilizing the bioreceptor on a channel or gate electrode (Figure 3d,e). There are several types of EGTs, including organic EGTs,^[^ ^98^, ^99^ ^]^ graphene EGTs,^[^ ^100^, ^101^ ^]^ nanowire EGTs,^[^ ^102^, ^103^ ^]^ and nanotubes EGTs.^[^ ^104^, ^105^ ^]^

FET biosensors have seen significant progress in recent years, and various structures and types of transistors have been used in biosensing applications. Regardless of the transistor adopted to develop the biosensor, the transistor preparation process significantly affects the characteristics and performance of the device. Moreover, the structural design of the transistor, including the channel length and width, gate dielectric layer thickness, and electrode material, plays an essential role in the sensitivity, selectivity, and response time of the biosensor. Additionally, the functionalization process of attaching a recognition element to the transistor surface determines the ability of the biosensor to sense analytes. Addressing these issues is critical for advancing the development of transistor‐based biosensors and enabling their widespread use in various applications.

## 4 Functionalization of Transistors for Biosensors

To achieve optimized performance in transistor‐based biosensors, the selection of a suitable functionalization method is crucial and depends on the electrode or channel material and the sensing principle of the transistor.^[^ ^106^ ^]^ However, not all functionalization methods facilitate the recognition of signal transduction. Notably, Debye screening can occur in both the electrolyte solutions and semiconductor regions, which can affect the performance of the biosensor.^[^ ^53^, ^73^, ^107^ ^]^ Moreover, the specificity and stability of the sensing interface composed of the bioreceptor substrate are influenced by several factors, including anti‐interference, accessibility of the target molecule, steric hindrance, and the applied electric field.^[^ ^108^, ^109^, ^110^, ^111^, ^112^ ^]^ These factors can affect the ability of a biosensor to detect a target analyte accurately and reliably. Therefore, careful consideration of these factors is essential when selecting functionalization methods and designing biosensors for specific applications.

### 4.1 Sensor Surface Immobilization Strategy

**Figure 4:**
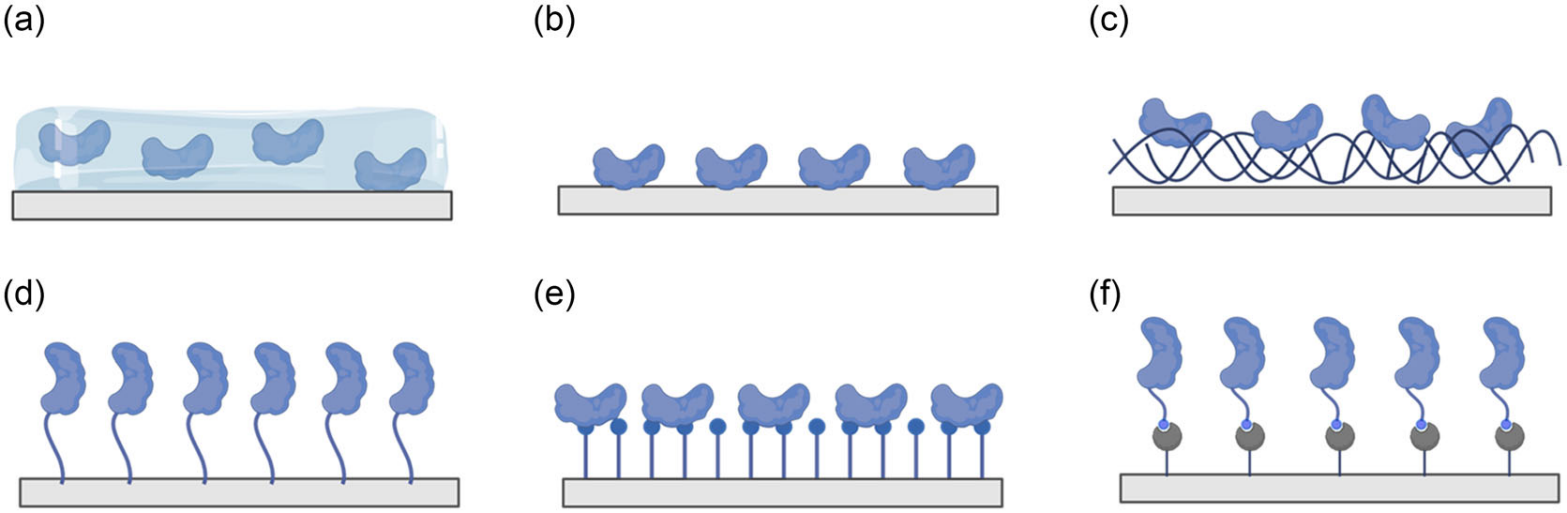
Common methods of immobilizing bioreceptors onto a substrate surface: a) entrapment; b) adsorption; c) cross‐linking; d) covalent attachment; e) self‐assembled monolayer; and f) affinity immobilization.

Immobilization is a standard method for functionalizing biosensors and involves making the biochemical components insoluble and fixing them to an appropriate interface (solid support or transducer surface) for separation, incubation, cleaning, and final detection.^[^ ^113^ ^]^ The choice of method depends on several factors, including the type of bioreceptor, nature of the target analyte, and the characteristics of the sensing surface. Entrapment, adsorption, cross‐linking, covalent attachment, self‐assembled monolayers (SAMs), and affinity immobilization are a few commonly used methods for immobilization, as shown in **Figure** **4**.^[^ ^49^, ^113^ ^]^

Entrapment refers to the encapsulation and immobilization of biomolecules in the 3D space of a polymer, to which attention needs to be paid: 1) the stability of the bioactivity of the encapsulated bioreceptor; 2) diffusivity of the target analyte within the matrix; and 3) effective coupling to and reliable transduction by FET.^[^ ^114^, ^115^ ^]^ Bioreceptors can also be immobilized on the surface of the substrate by physical adsorption or ion binding, which is called adsorption. Physical adsorption is a reversible process, and the use of sparsely porous or polar materials as carriers or pretreatment of the solid‐phase surface to increase the charge can promote adsorption and reduce desorption.^[^ ^116^ ^]^ Bioreceptors can also be immobilized by forming a covalent linkage with functional groups on the substrate surface through a crosslinking agent, forming a crosslinked mesh structure. For example, glutaraldehyde (GA) contains aldehyde groups at both ends, which help to implement a spatial network between proteins by forming Schiff bases with the free amino groups of the protein molecules.^[^ ^117^ ^]^ Covalent attachment involves the immobilization of a bioreceptor with a linker molecule on the substrate surface. This method allows the bioreceptor to attach firmly and stably to the substrate surface by covalently modifying the linker and is relatively straightforward to manipulate.^[^ ^118^ ^]^ SAMs are ordered molecular arrangements spontaneously formed on the surfaces of various substrates. These 2D molecular structures are highly oriented and created through a “bottom‐up” process, allowing for the creation of novel molecular architectures that can be tailored to specific applications.^[^ ^119^ ^]^ SAMs of organic disulfides, alkanethiols, and sulfides are strongly adsorbed onto different metals and transition metal dichalcogenides (TMDCs).^[^ ^120^, ^121^ ^]^ The rearrangement and reorientation of SAMs adsorbed on the Au surface by the Au–S bond occurred slowly (10–20 h), resulting in a uniform, molecularly arranged, densely packed, and low‐defect cover layer.^[^ ^122^ ^]^ The sensitivity of detection is improved by precise control of the interlayer distance, improved molecular orientation of the immobilized bioreceptor, and surface density.^[^ ^123^ ^]^ The combination of biopolymers with a solid support is achieved by natural receptors, providing particular and durable binding of the counterpart.^[^ ^113^ ^]^ Biotin and avidin (or streptavidin) have very high affinities for each other, and their binders are stable and almost independent of the temperature, pH, and concentration. In addition, one affinity molecule (streptavidin) can bind to four biotin molecules, improving the efficiency of bioreceptor immobilization. A commonly used method is to modify streptavidin on the surface of substrates, followed by the immobilization of biotin‐labeled bioreceptors.^[^ ^124^ ^]^ In conclusion, maintaining the activity of immobilized bioactive compounds and reducing their specific adsorption are essential research questions. Anchorage firmness, distribution uniformity, and effective area of the bioreceptor affect the durability, noise, and sensitivity of the sensor. Therefore, careful consideration of the immobilization method and optimization of the immobilization conditions are essential for obtaining stable and sensitive biosensor performance.

### 4.2 Surface Immobilization on Gold

**Figure 5:**
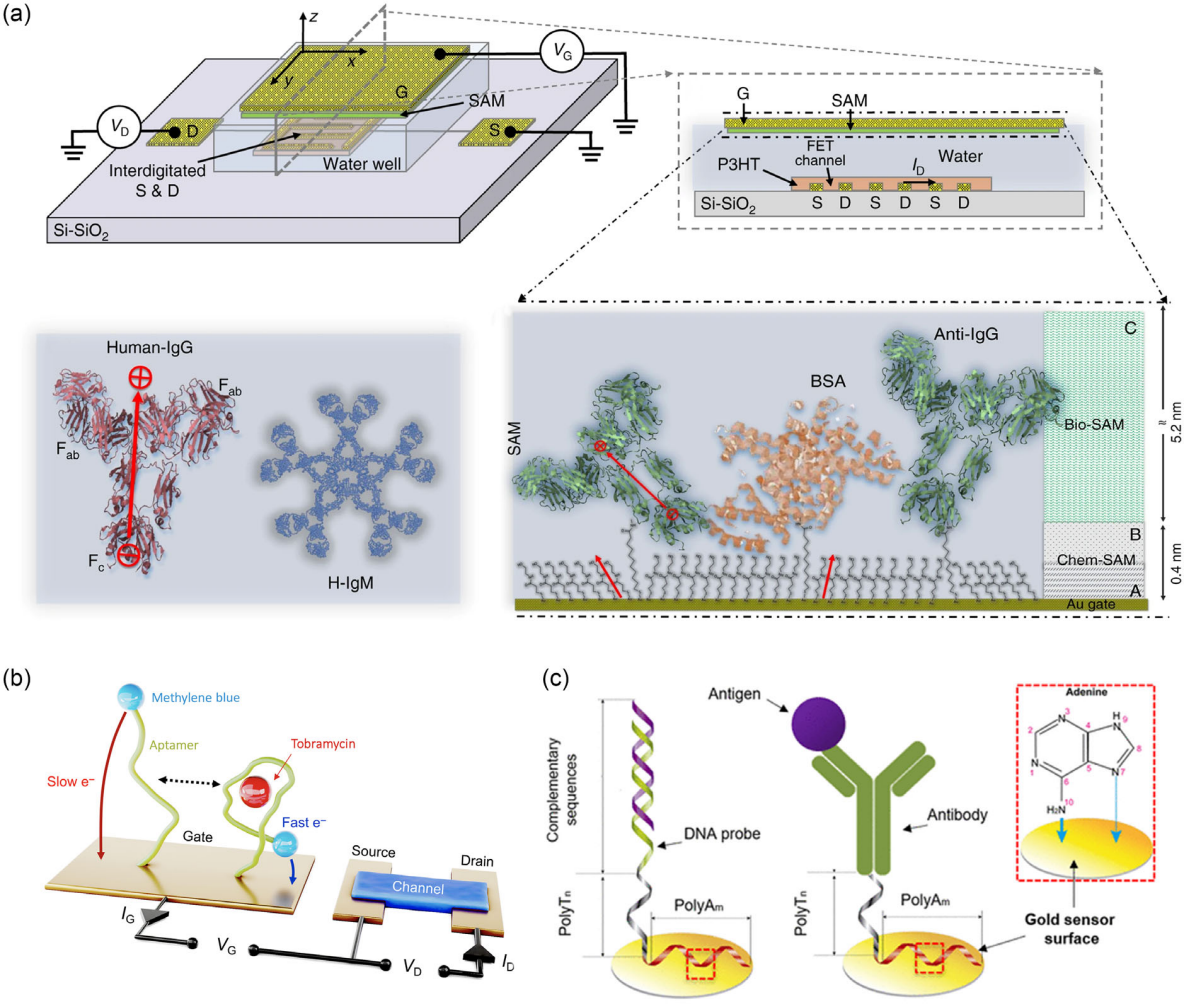
Immobilization of bioreceptors on Au surfaces. a) Structural diagram of a FET comprising a gold gate modified with a self‐assembled monolayer (SAM) of the capturing anti‐IgGs. Reproduced under the terms of the CC‐BY Creative Commons Attribution 4.0 International license (https://creativecommons.org/licenses/by/4.0).^[^ ^73^ ^]^ Copyright 2018, The Authors, published by Springer Nature. b) Structural diagram of an OECT with the immobilization of aptamer on Au gate via Au‐S bond. Reproduced under the terms of the CC‐BY Creative Commons Attribution 4.0 International license (https://creativecommons.org/licenses/by/4.0).^[^ ^134^ ^]^ Copyright 2022, The Authors, published by the American Association for the Advancement of Science. c) The nucleic acid probe and antibody were immobilized on the Au surface via the adsorption affinity of polyAm. Reproduced with permission.^[^ ^136^ ^]^ Copyright 2020, American Chemical Society.

Gold is commonly used as a substrate for immobilizing transistor‐based biosensors because of its unique properties, including high conductivity, biocompatibility, ease of patterning, and inertness. There are several common methods for immobilizing bioreceptors on gold surfaces, including SAMs, covalent attachment, and adsorption. Torsi et al.^[^ ^73^ ^]^ reported a SAM‐based antibody immobilization method on the gold gate of an OFET to achieve single‐molecule detection (**Figure** **5**a). The mixed alkanethiols with carboxylic terminal groups (3‐mercaptopropionic acid (3‐MPA) and 11‐mercaptoundecanoic acid (11‐MUA), 10:1) self‐assembled on the cleaned Au surface via the interaction of sulfhydryl groups and Au to form Au‐S bonds, forming a chemical SAM (chem‐SAM). The carboxylic group was then activated by *N*‐(3‐dimethylaminopropyl)‐*N*′‐ethylcarbodiimide hydrochloride (EDC) and *N*‐hydroxysuccinimide (NHS) and conjugated to the amino group of the antibodies to achieve bioreceptor immobilization. The unreacted sulfo‐NHS groups were further treated with 1 m ethanolamine in a process known as “chemical blocking”. The Au electrodes were immersed in (0.1 mg mL^−1^) BSA solution to achieve “bio‐blocking”. The immobilization process for building a sensing layer on the surface of the Au gate can be summarized in three steps: 1) self‐assembly of linker‐bearing molecules on the gold surface; 2) anchoring of the bioreceptor to the self‐assembled membrane for specific recognition and binding of the target analyte; and 3) passivation treatment with an (antifouling) material that minimizes nonspecific adsorption, thereby ensuring high selectivity and sensitivity of the biosensor.^[^ ^125^ ^]^ This strategy has been widely adopted in the construction of electrolyte‐gated transistor biosensors owing to its simplicity, reliability, and compatibility with a range of bioreceptors.^[^ ^71^, ^74^, ^93^ ^]^

Single‐stranded oligonucleotide probes are usually prepared using synthetic methods and modified with linkers at the 5′ or 3′ end for immobilization on the Au electrode surface via Au‐S bond, such as thiolated capture probes (SHCP).^[^ ^126^ ^]^ Oligonucleotide probes can be immobilized by alkanethiol linkers,^[^ ^127^, ^128^ ^]^ cyclic disulfides (SS),^[^ ^129^ ^]^ or multiple disulfide linkers.^[^ ^130^ ^]^ In addition, because of the base‐dependent adsorption of single‐stranded DNA (ssDNA) on gold surfaces,^[^ ^131^ ^]^ it is necessary to passivate the surface using molecules with polar OH headgroups, such as 6‐mercapto‐1‐hexanol (MCH). MCH passivation is an effective method to disrupt base attachment because of its high affinity for gold.^[^ ^132^ ^]^ This passivation step ensures that the thiolated oligonucleotide probe is oriented upright and extends toward the solution while minimizing nonspecific adsorption.^[^ ^133^ ^]^ In a recent study, Malliaras et al.^[^ ^134^ ^]^ developed a novel method for immobilizing ssDNA aptamers on gold gates of organic electrochemical transistors (OECTs), as shown in Figure 5b. To enhance the sensitivity of tobramycin detection, a square‐wave voltage (SWV) operation was employed at the gate electrode. Specifically, a 2 μL aliquot of 100 μm aptamer with a thiol linker (–HO–(CH2)6‐S‐S‐(CH2)6‐) in 1× phosphate‐buffered saline (PBS) was thawed and mixed with 4 μL of 10 mm tris(2‐carboxyethyl)phosphine (TCEP) for 8 h to reduce the disulfide bond. Subsequently, the aptamer solution was diluted to 500 nm with PBS and incubated overnight on a cleaned gold surface. Next, the electrodes were rinsed and placed in 10 mm MCH for 5 h to passivate them and rinsed again with PBS. The gate was activated by plasma treatment before ssDNA incubation. The covalent attachment of bioreceptors with modified linkers to gold surfaces (or gold nanoparticles)^[^ ^72^, ^135^ ^]^ is a common and effective method. However, further studies are required to optimize the performance of these biosensors, including the selection of linkers, surface passivation, and detection methods. Direct adsorption of bioreceptors on gold surfaces is an excellent alternative.

An innovative immobilization method utilizes the remarkable affinity of the polyadenine (polyA) sequence for gold surfaces (the relative adsorption affinity of DNA bases is A > C  ≥ G > T on Au surfaces).^[^ ^131^ ^]^ Huertas et al.^[^ ^136^ ^]^ reported a method in which polyAm‐polyTn tails were incorporated into nucleic acid probes and antibodies for direct immobilization on gold surfaces (Figure 5c). This adsorption is attributed to the coordination of the metal to the N atoms of the amine group and the N7 atom. Supported lipid bilayers (SLBs) loaded with bioreceptors adsorbed on the Au surface via hydrophilic interactions are promising functionalization methods.^[^ ^137^ ^]^

### 4.3 Surface Immobilization on Silicon‐Based Materials

**Figure 6:**
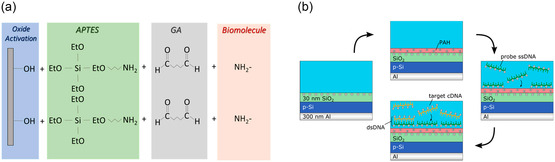
Immobilization of bioreceptors on silicon‐based material surfaces. a) A schematic view of APTES protocol for biomolecule immobilization. Reproduced under the terms of the CC‐BY Creative Commons Attribution 4.0 International license (https://creativecommons.org/licenses/by/4.0).^[^ ^145^ ^]^ Copyright 2022, The Authors, published by MDPI. b) A schematic of the PAH layer preparation, electrostatic adsorption of probe ssDNA molecules onto the gate surface, and subsequent hybridization with cDNA molecules. Reproduced with permission.^[^ ^116^ ^]^ Copyright 2015, American Chemical Society.

Silicon is a metalloid with a crystalline structure widely used in the semiconductor and electronics industries. Si‐based materials used for transistor biosensing include silicon wafers, silicon nanowires (SiNWs), silicon dioxide (SiO_2_), and silicon nitride (SiN_*x*_). Upon exposure to air, the silicon surface underwent spontaneous oxidation, forming a thin amorphous layer of silicon oxide. The oxide layer can be used as a modified functional interface, allowing immobilization of bioreceptors.^[^ ^138^, ^139^ ^]^ Silanization is the most common method for anchoring bioreceptors on silicon‐based material surfaces through the interaction of chemically active hydroxyl groups with a silane grafting agent to form a siloxane network structure containing functional groups.^[^ ^140^ ^]^ This technique can also be applied to a variety of substrates, some with hydroxyl groups of their own, and others that can be introduced through various methods such as KOH/NaOH, acid, piranha solution, or plasma treatment. Biomolecule anchoring can be achieved using different cross‐linkers.^[^ ^92^, ^141^, ^142^, ^143^ ^]^ All metals apart from the noble metals are covered by a native oxide layer with a thickness of only several nanometers under atmospheric conditions. These passive oxide layers were terminated by hydroxyl groups and covered with an adsorbed water layer to minimize the surface potential.^[^ ^117^ ^]^ A commonly used silanization reagent, 3‐aminopropyltriethoxysilane (APTES), introduces amine groups that covalently bind to various biomolecules, such as DNA and proteins.^[^ ^91^ ^]^ APTES reacts with the free hydroxyl groups of the oxidized substrate via SN_2_ exchange with ethanol loss.^[^ ^117^ ^]^ Ko et al.^[^ ^144^ ^]^ reported a method for immobilizing prostate‐specific antigen antibodies (anti‐PSA) on a polycrystalline silicon nanowire field‐effect transistor (poly‐Si NWFET). The FET sensor was cleaned in a mixture of ethanol and acetone (1:1) and the poly‐Si nanowires were modified using APTES. The GA was used as a cross‐linker, and the aldehyde groups at both ends were bonded to the amino group on the substrate surface and the free amino group of the antibody, respectively. **Figure** **6**a shows a schematic diagram of the APTES protocol used for biomolecule immobilization.^[^ ^145^ ^]^ This method is also applicable to the immobilization of amino‐modified ssDNA,^[^ ^145^, ^146^ ^]^ where the introduced carbon chain (C_6_) increases the spatial distance, preventing the binding of the amino group of the base to the aldehyde group, and ensuring an upright state of the ssDNA probe. Gupta et al.^[^ ^147^ ^]^ reported another method to produce high‐density amino groups on a SiNx surface using hydrogen gas as the reactive medium and inductively coupled plasma reactive ion etching (ICP‐RIE).

The electrostatic adsorption of bioreceptors is another method for functionalizing surfaces, which is simple, rapid, and suitable for a wide range of substrate shapes and forms.^[^ ^148^ ^]^ Due to the negative charge of DNA, substrate/cationic‐polyelectrolyte/ssDNA layer‐by‐layer (LbL) electrostatic adsorption has become a popular method for nucleic acid immobilization.^[^ ^116^, ^149^, ^150^, ^151^ ^]^ For example, poly‐l‐lysine (PLL)‐modified silicon field‐effect sensors for DNA detection,^[^ ^150^ ^]^ PLL‐modified graphene field‐effect transistor (GFET) for RNA detection,^[^ ^149^ ^]^ and poly(allylamine hydrochloride) (PAH)‐modified field‐effect sensors for DNA detection (Figure 6b).^[^ ^116^ ^]^

### 4.4 Surface Immobilization on Carbon Nanomaterials

**Figure 7:**
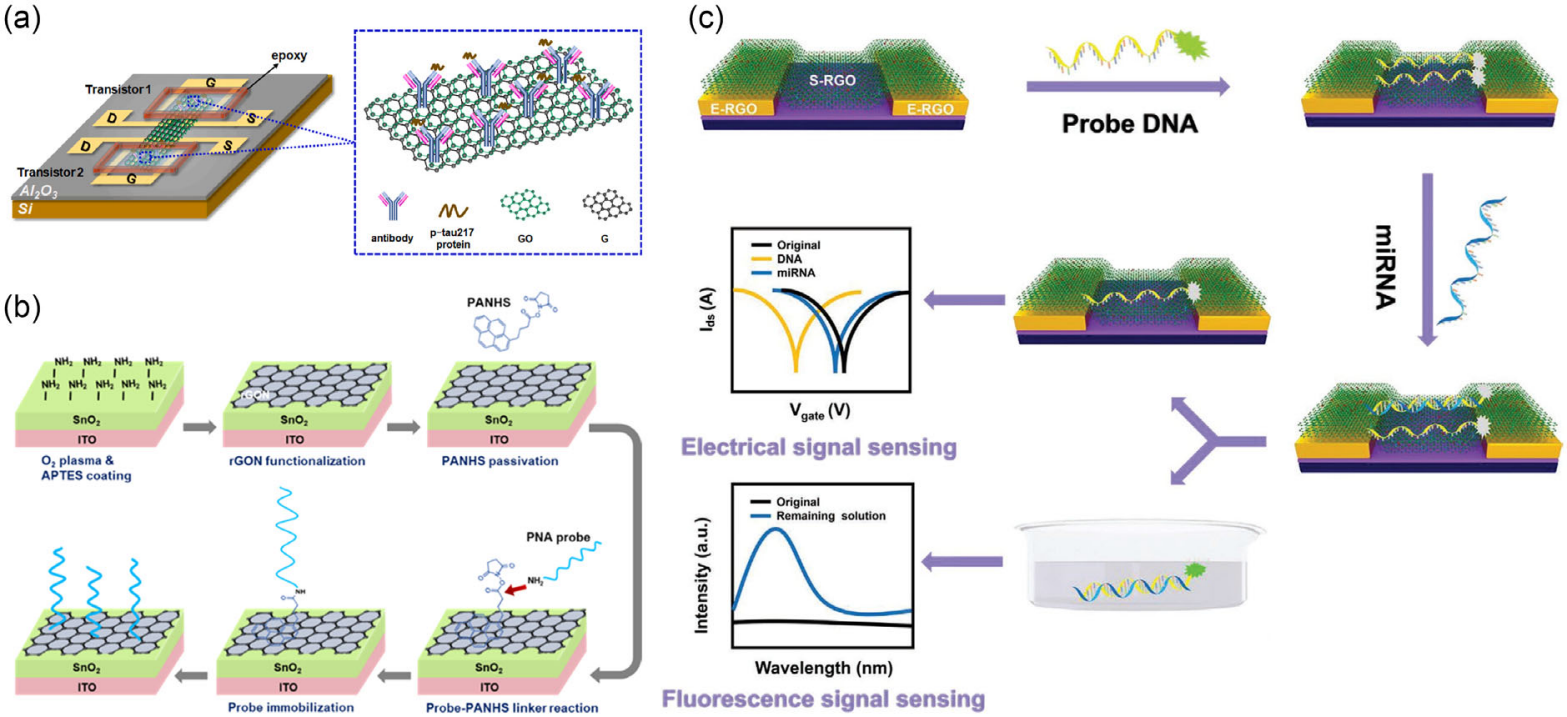
Immobilization of bioreceptors on graphene material surfaces. a) A schematic image of a GFET with antibodies covalently bound to GO/G surface. Reproduced with permission.^[^ ^158^ ^]^ Copyright 2023, Elsevier. b) Chemical fabrication and surface modification of the biosensor chip. PANHS was immobilized on rGO surface via *π*–*π* stacking for anchoring the PNA probe. Reproduced with permission.^[^ ^160^ ^]^ Copyright 2021, American Chemical Society. c) Sensing mechanism of the dual‐signal biosensor, and GO showed a preference for adsorbing ssDNA over dsDNA. Reproduced with permission.^[^ ^164^ ^]^ Copyright 2020, Wiley‐VCH.

Graphene and carbon nanotubes (CNTs) are popular carbon materials used in transistor‐based biosensors. Graphene is a 2D nanomaterial consisting of a single‐carbon atomic layer arranged in a honeycomb lattice.^[^ ^152^ ^]^ This is the basic structure of many other carbon isomers such as graphite and CNTs.^[^ ^153^ ^]^ Graphene has high electrical conductivity, a large surface area, excellent mechanical strength, easy functionalization, chemical stability, biocompatibility, and sensitivity to the surrounding electric field and charges, making it an ideal substrate for immobilizing bioreceptors and detecting biomolecules.^[^ ^100^ ^]^ Covalent functionalization involves covalent bonding between the functional groups on the surface of graphene and bioreceptors. One common approach is the use of organic diazonium salts, which involves a reaction between the reactive radical and aromatic systems of the graphene sheet to form covalent bonds.^[^ ^154^, ^155^ ^]^ Graphene oxide (GO) and reduced graphene oxide (rGO) are graphene derivatives containing various oxygen‐containing groups, such as carboxyl, carbonyl, and hydroxyl. These functional groups provide reactive sites for covalent functionalization via substitution reactions.^[^ ^156^, ^157^ ^]^ For example, Huang et al.^[^ ^158^ ^]^ reported a method of antibody‐functionalized solution‐gated field effect transistor (SGFET) for phosphorylated tau‐217 protein (p‐tau217) detection (**Figure** **7**a). The antibody was immobilized on the GO/graphene surface via a carbodiimide‐mediated reaction between the amine groups of the antibody and the carboxyl groups of GO.

In contrast, the noncovalent functionalization of graphene involves the *π*–*π* or hydrophobic stacking, electrostatic interaction, and van der Waals interaction. This method provides a straightforward and mild approach for functionalization and can fully preserve the lattice and electronic properties of graphene.^[^ ^154^, ^159^ ^]^ One common approach for noncovalent functionalization is the use of *π*–*π* stacking between the aromatic rings of the bioreceptor and the graphene surface. The collective effect of multiple *π*–*π* stacking interactions can result in a strong and stable binding between the bioreceptor and graphene surface. For instance, 1‐pyrenebutyric acid *N*‐hydroxysuccinimide ester (PBASE) is a biofunctional linker molecule that can bind to the basal plane of graphene through the noncovalent *π*–*π* interaction of the aromatic pyrenyl group. Subsequently, the bioreceptor (protein or DNA) containing the amino terminus was covalently bound to PBASE via *N*‐hydroxysuccinimide (NHS) cross‐linking reactions and immobilized on graphene.^[^ ^160^, ^161^, ^162^, ^163^ ^]^ As shown in Figure 7b, Lee et al.^[^ ^160^ ^]^ reported a method for noncovalent functionalization on the extended‐gate surface. The reduced graphene oxide nanosheet (rGON) was deposited on amino‐rich SnO_2_ surface, and the pyrenebutyric acid N‐hydroxy succinimide ester (PANHS or PBASE) was modified on the rGON surface via *π*–*π* interaction and anchored the PNA probe for miRNA detection. In another study, Liu et al.^[^ ^164^ ^]^ reported a method to functionalize all‐graphene‐based FETs (AG‐FETs) for miRNA detection by adsorbing ssDNA probes on the GO surface (Figure 7c). The underlying mechanism is based on the fact that GO has a preference for adsorbing ssDNA over double‐stranded DNA (dsDNA) owing to the disruption of *π*–*π* stacking interactions.^[^ ^165^ ^]^ The functionalization of CNTs for biosensing applications is similar to that of graphene, involving both covalent and noncovalent methods. The same chemical reactions can be used for CNT functionalization, which involves the chemical attachment of functional groups, such as carboxyl, amine, and thiol groups, that react with the complementary groups present on the biomolecules, thus forming covalent bonds.^[^ ^166^ ^]^

### 4.5 Surface Immobilization on Organic Semiconductor Materials

**Figure 8:**
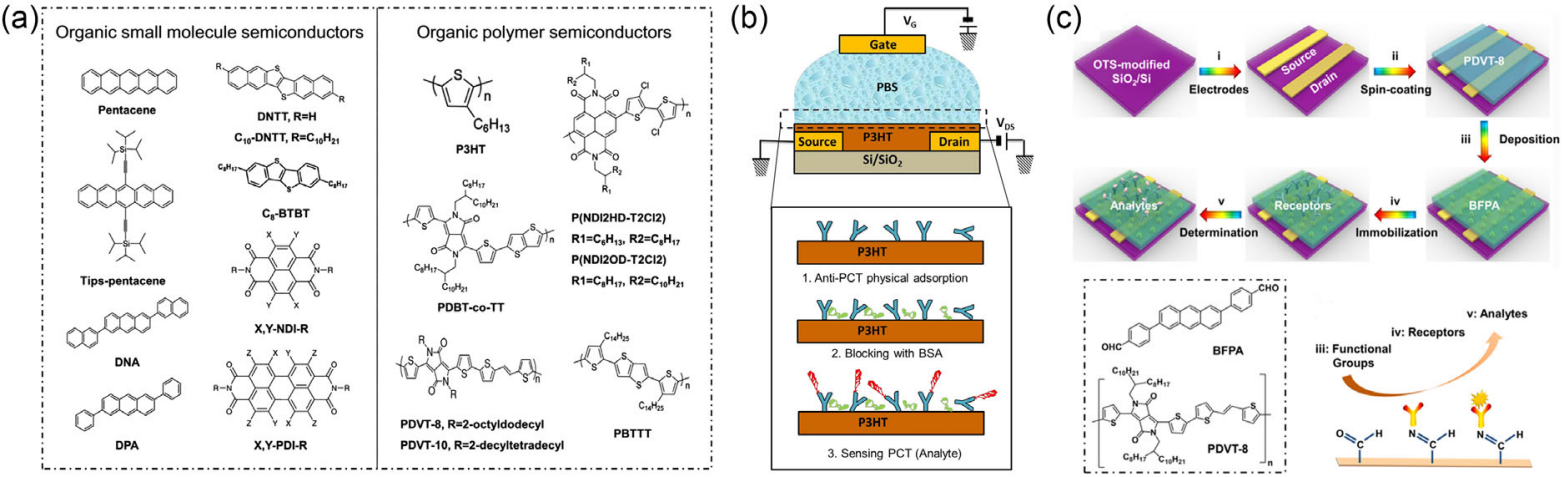
Immobilization of bioreceptors on organic semiconductor material surfaces. a) Chemical structures of common organic semiconductors (OSCs). Reproduced under the terms of the CC‐BY Creative Commons Attribution 4.0 International license (https://creativecommons.org/licenses/by/4.0).^[^ ^98^ ^]^ Copyright 2021, The Authors, published by The Hong Kong Polytechnic University and John Wiley & Sons Australia, Ltd. b) A diagram of the OFET‐based biosensor designed for procalcitonin (PCT) detection. Anti‐PCT were immobilized on the surface of the polymer poly(3‐hexylthiophene) (P3HT) via physical adsorption. Reproduced with permission.^[^ ^173^ ^]^ Copyright 2017, Elsevier. c) The design and principle of the OFET‐based AFP biosensors. Reproduced with permission.^[^ ^175^ ^]^ Copyright 2021, American Chemical Society.

High‐performance polymers and small‐molecule organic semiconductor (OSC) materials have been researched and developed as potential biofunctionalized materials (**Figure** **8**a). Conjugated OSCs have good biocompatibility and ease of functionality owing to their chemical structure, which is similar to that of various biological substances^[^ ^167^ ^]^ and weak interactions with external substances, ensuring stable sensing of chemical and biological parameters.^[^ ^98^ ^]^ OSCs have attracted significant attention for biomedical applications.^[^ ^168^ ^]^ Moreover, OFETs have potential advantages for biosensor applications owing to their high carrier mobility, large on/off ratio, low threshold voltage, steep subthreshold swing, and good stability.^[^ ^98^, ^169^ ^]^ The physical adsorption of bioreceptors on the surface of an OSC layer is a common approach for preparing OFET‐based biosensors. The bioreceptor is attached to the surface through weak interactions such as van der Waals forces, hydrogen bonding, or electrostatic interactions. Physical adsorption is a simple and convenient method that does not require chemical modification of the surface or the bioreceptor. Ditte et al.^[^ ^170^ ^]^ synthesized a triblock copolymer (TBC) consisting of a conjugated polydiketo‐pyrrolopyrrole‐thienothiophene (PDPP‐TT) polymer‐conjugated semiconductor (PSC) flanked by two soft elastic poly(dimethylsiloxane) (PDMS) segments. PDMS is a flexible biocompatible polymer.^[^ ^171^ ^]^ SARS‐CoV‐2 spike RBD receptors and S1 Abs were directly adsorbed onto TBC. Spin‐coating is another method used to adsorb bioreceptors onto a semiconductor layer.^[^ ^172^ ^]^ The spin‐coating speed affects the diffusion of the bioreceptor in the film, thereby affecting the performance of the biosensor. Torsi et al.^[^ ^173^ ^]^ developed an electrolyte‐gated OFET immunosensor for detecting procalcitonin. The capturing antibodies were immobilized on the surface of P3HT through direct physical adsorption without pretreatment (Figure 8b). Cheng et al.^[^ ^174^ ^]^ demonstrated a high‐performance OFET biosensor based on the conjugated polymer PDBT*‐*co‐TT for the detection of alpha‐fetoprotein (AFP). Anti‐AFP antibodies were immobilized on the PDBT*‐*co‐TT surface by physical adsorption. However, probes deposited by physical adsorption have poor uniformity and relatively weak interactions with the surface of the functional layer of the device, which affects its long‐term stability. The introduction of functional groups onto the surface of semiconductor materials for covalent attachment to bioreceptors should be considered. The unpredictable degradation of OFET performance due to the complex synthesis and chemical functionalization processes is a challenge. Therefore, Cheng and co‐workers^[^ ^175^ ^]^ proposed a new covalent strategy in another report. In this study, an OFET‐based biosensor modified with 2,6‐bis(4‐formylphenyl)anthracene (BFPA) materials for the detection of AFP was reported (Figure 8c). BFPA plays a role in maintaining the device performance and covalently binds bioreceptors.

However, organic materials directly exposed to solutions are susceptible to interference from other ions, which reduces the selectivity and stability of the sensor. In addition, testing remains difficult for analytes that do not carry an electrical charge in solution. Molecularly imprinted polymers (MIPs) operate by a “lock and key” mechanism to selectively bind the molecule with which they were templated during production.^[^ ^176^ ^]^ Compared to antibodies, MIPs are not only specific to the target analyte but are also more stable and reproducible.^[^ ^177^ ^]^ Parlak et al.^[^ ^67^ ^]^ developed an OECT to achieve selective sensing of cortisol in human sweat. An MIP‐based artificial recognition membrane was interposed between the PEDOT:PSS channel layer and analyte (sweat) reservoir. In this study, specificity of the sensor was achieved by constructing a molecular template using copolymerized functional monomers and cross‐linkers in the presence of an analyte. The sensor's sensing principle relies on the utilization of sealed MIP‐based selective membrane pores in the presence of the analyte, which prevents ion movement into the channel and reduces the channel current of the OECT. The MIP functionalization strategy offers significant advantages over natural receptors in terms of durability and low cost, allowing for applications over a wide temperature range and indefinite storage. This provides an ideal general platform for biodetection in the applicable range and enables better integration into electronics.

### 4.6 Nonimmobilized Secondary Sensing Strategy

It is worth mentioning that the immobilization of bioreceptors is not always necessary for transistor‐based biosensing. ISFETs and GFETs are examples of transistor‐based biosensors sensitive to hydrogen ions. This sensitivity allows the transduction and amplification of pH changes resulting from biochemical reactions in the electrolyte into electrical signals. Loop‐mediated isothermal amplification (LAMP) technology has been used in transistor‐based biosensors owing to the nature of the reaction process, which changes the pH of the solution. Target nucleic acid sequences can be detected indirectly based on the sensor's high sensitivity to pH. The specificity of detection is determined by the ability of LAMP to recognize the target sequence.^[^ ^178^, ^179^ ^]^ The nonimmobilization method in transistor‐based biosensors simplifies the biofunctionalization process and reduces costs, providing label‐free, real‐time monitoring of biochemical reactions, which promises a universal platform for biosensing.

## 5 Application in SARS‐CoV‐2 Detection

### 5.1 Detection of SARS‐CoV‐2 Spike Protein

One vital encoded structural protein of SARS‐CoV‐2 is the spike glycoprotein (S), which consists of three S1‐S2 heterodimers that bind to angiotensin‐converting enzyme 2 (ACE2) receptors on type II pneumocytes.^[^ ^180^ ^]^ Spike proteins elicit an earlier immune response than other viral proteins and have two critical functions: receptor recognition and membrane fusion.^[^ ^181^, ^182^ ^]^ During the process of viral invasion, the S1 subunit of the spike protein binds to host cell surface receptors to facilitate viral attachment, whereas the S2 subunit promotes the fusion of host and viral cell membranes, allowing for viral gene entry into the host cell.^[^ ^183^ ^]^ As a result, the detection of the presence of spike proteins has become a significant target for diagnosing SARS‐CoV‐2 infections. The immune system produces specific antibodies when the individual is infected with the SARS‐CoV‐2 virus. The recognition of antigens and antibodies provides a stable specificity for the detection of viruses via biosensors. Another method involves the use of the functional receptor ACE2 of the SARS‐CoV‐2 virus.^[^ ^184^ ^]^ The spike protein of the virus mediates its entry into host cells by binding to ACE2.^[^ ^185^, ^186^ ^]^ In comparison to the first spike protein of the SARS coronavirus, the spike protein of SARS‐CoV‐2 possesses a higher affinity for ACE2.^[^ ^187^ ^]^ Thus, ACE2 is considered a specific probe for detecting the SARS‐CoV‐2 spike protein, offering an alternative approach to antibody‐based biosensors. A range of transistor‐based biosensors with high sensitivity and specificity have been developed for the detection of spike proteins. This makes them promising candidates for developing rapid and accurate diagnostic tests for SARS‐CoV‐2 infections.

**Figure 9:**
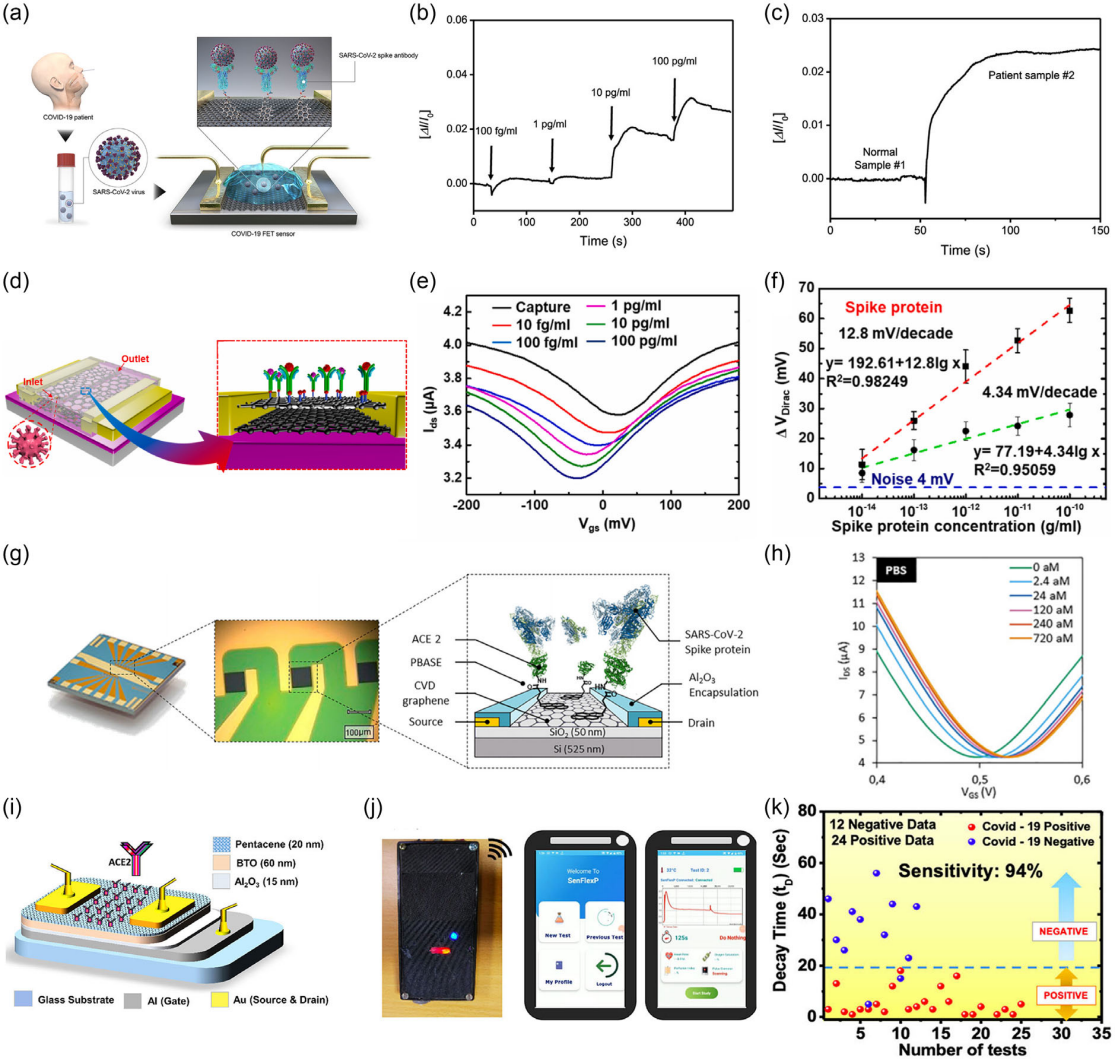
Transistor‐based biosensors for SARS‐CoV‐2 spike protein detection. a) A schematic of the FET biosensor for detection of SARS‐CoV‐2 virus from the individual. b) Real‐time response of FET biosensor toward SARS‐CoV‐2 antigen protein in UTM. c) Response signal between normal and patient samples. a–c) Reproduced with permission.^[^ ^40^ ^]^ Copyright 2020, American Chemical Society. d) A schematic structure and protein detection illustration of the GO/Gr heterostructure FET biosensor. e) The transfer curves of the GO/Gr heterostructure FET biosensor. f) The protein concentration of sensor GO/Gr (red line) and sensor Gr (blue line) depends on the relationship between the variation of Δ*V*_Dirac_. d–f) Reproduced with permission.^[^ ^192^ ^]^ Copyright 2022, Elsevier. g) The diagram of GFET array biosensor. h) The transfer curve of the GFET. g,h) Reproduced with permission.^[^ ^194^ ^]^ Copyright 2023, Royal Society of Chemistry. i) A schematic design of the FET biosensor. j) A schematic diagram of SenFlex. k) A plot of decay time as a function of the number of tests. i–k) Reproduced with permission.^[^ ^172^ ^]^ Copyright 2022, American Chemical Society.

Graphene field‐effect transistors (GFETs), which use graphene as the channel material, have been widely used in biosensing because of their easy functionalization and good electrical properties.^[^ ^188^ ^]^ Seo et al.^[^ ^40^ ^]^ fabricated a label‐free SARS‐CoV‐2 spike protein FET biosensor by immobilizing the SARS‐CoV‐2 spike antibody on the graphene channel, as shown in **Figure** **9**a. The PBASE acted as the linker attached to the graphene surface by *π*–*π* stacking and covalently bound to the antibody at the other end. Detection of SARS‐CoV‐2 based on the antibody and the protein interactions. The LOD for spike protein was found to be 1 fg mL^−1^ in PBS solution and 100 fg mL^−1^ in clinical transport medium (Figure 9b). In addition, it was also demonstrated that the SARS‐CoV‐2 of clinical nasopharyngeal swab samples and culture medium could also be detected, with a LOD of 2.42 × 10^2^ copies mL^−1^ and 1.6 × 10^1^ pfu mL^−1^, respectively. The sensor could clearly distinguish normal and patient samples (Figure 9c), showing potential for clinical application.

SARS‐CoV‐2 evolution has been characterized by the emergence of new variants carrying immune escape mutations,^[^ ^189^ ^]^ which reduce the binding affinity to diagnostic antibodies. In contrast, binding affinity for the ACE2 receptor is enhanced when certain mutations occur.^[^ ^190^ ^]^ Therefore, the use of ACE2 as a biosensor probe is considered a rational choice. Romagnoli et al.^[^ ^191^ ^]^ developed a POC device to detect SARS‐CoV‐2 spike proteins. The device is based on a GFET‐based biosensor system with 12 separate monolayer graphene channels. The authors used a computer‐assisted protein design method to link the extracellular portion of the ACE2 receptor to the fragment‐crystallizable (Fc) domain of immunoglobulins. This approach generated a stable ACE2‐Fc chimera in the absence of a cell membrane, which was then immobilized on graphene using PBASE to recognize spike proteins. The LOD was 20 pg mL^−1^ in PBS. Moreover, the sensor was used to detect viral mutant strains, successfully achieving specific identification of nasopharyngeal swab samples.

As research related to GFETs progresses, various graphene‐derived materials are emerging and being applied in biosensors. Gao et al.^[^ ^192^ ^]^ first fabricated a biosensor for detecting the SARS‐CoV‐2 spike protein, which consisted of a graphene oxide‐graphene (GO/Gr) stacking graphene van der Waals heterostructure FET integrated with a microfluidic chip (Figure 9d). The GO/Gr heterostructure was in situ formed through *π*–*π* stacking. Testing of the Dirac point revealed that the transistor with this structure had 1.5 times higher mobility than the single‐layer graphene transistor. The higher mobility was due to the inhibitory effect of graphene oxide on surface ion absorption, and the interface coupling of the heterojunction improved the efficiency of electron exchange, which effectively improved the performance of the device. The captured antibodies were modified on the graphene oxide through *π*–*π* interactions. The channel potential changed when the antibody reacted with the SARS‐CoV‐2 spike protein, leading to a shift in the Dirac point (Figure 9e). The sensor had a response time of less than 20 min with an LOD of 8 fg mL^−1^ and an average sensitivity of 12.8 mV decade^−1^, whereas the sensitivity of the Gr‐FET decreased to 4.76 mV decade^−1^ (Figure 9f).

When detecting in an electrolyte, the sensitivity of the sensor should consider the effect of the Debye length (*λ*_D_) of the electric double layer. The *λ*_D_ is a measure of the screening length of charged particles in the electrolyte and can affect the efficiency of the sensor's detection mechanism. Reactions occurring outside of the *λ*_D_ will be screened, leading to a reduced contribution to the sensor's sensitivity. Thus, the detection mechanism should be designed to take into account the *λ*_D_, ensuring that bioreceptors are located within this range for maximum sensitivity.^[^ ^193^ ^]^ Silvestri et al.^[^ ^194^ ^]^ fabricated a GFET‐based biosensor array using ACE2 as the receptor. The biosensor array consisted of 12 CVD graphene devices modified with ACE2 via a PBASE linker (Figure 9g). By optimizing the thickness of the functionalized layer and the ionic strength of the electrolyte, the Debye screening effect was reduced. The transfer curve of the sensor as shown in Figure 9h showed that the LOD of the sensor could reach 2.94 aM in PBS.

CNTs are being promoted as a promising channel material for biosensing applications owing to their 1D structure and ultrathin body, which reduces short‐channel effects. CNTs are mainly divided into two groups: single‐walled carbon nanotubes (SWCNTs) and multiwalled carbon nanotubes (MWCNTs).^[^ ^195^ ^]^ Of these, high‐purity semiconducting s‐SWCNTs exhibit a high current‐carrying capacity.^[^ ^196^ ^]^ FET devices using this type of s‐SWCNT network exhibit excellent sensitivity for biosensing.^[^ ^197^ ^]^ Shao et al.^[^ ^198^ ^]^ modified s‐SWCNTs with a SARS‐CoV‐2 spike protein antibody to construct a sensor chip containing four FETs. The carboxylic acid groups of the s‐SWCNTs were activated by EDC and NHS and then covalently linked to the antibody. After the addition of different concentrations of SARS‐CoV‐2 spike protein, additional hole carriers in the s‐SWCNTs were induced owing to the negative charge from the proteins, which triggered a positive shift in the transfer curves. The obtained biosensor showed an LOD of 0.55 fg mL^−1^ and false‐negative rate of 17.8% compared with standard clinical tests.

Graphene‐based sensors often suffer from increased leakage current, which is caused by the zero bandgap of graphene.^[^ ^199^ ^]^ The adjustable bandgap of TMDCs gives it a better signal‐to‐noise ratio.^[^ ^200^ ^]^ WSe_2_ crystal, a semiconducting transition metal dichalcogenide, has shown great advantages for biosensing owing to its large surface area, strong absorption capacity, and high surface activity.^[^ ^201^ ^]^ The FET with monolayer WSe_2_ as a channel exhibits ultrahigh sensitivity. Hafshejani et al.^[^ ^121^ ^]^ used monolayer WSe_2_ as the sensing material and fabricated interdigitated electrode on its surface to establish a 2D‐FET biosensor. MUA was used as probe linker to immobilize the SARS‐CoV‐2 spike antibody on the surface of WSe_2_. In this case, MUA compensated for the Se vacancy and nearby dangling bonds, causing the p‐type behavior of the device to become weaker. After adding the antigen and antibody, the p‐type behavior increased slightly owing to the charge transfer processes. The LOD of the sensor reaches 25 fg μL^−1^ in 0.01 × PBS.

Transistors using 1D or 2D materials such as CNTs or graphene as the channel have inherent complexities in design and preparation, limiting large‐scale commercial applications, and probe functionalization usually requires chemical reactions. Mandal et al.^[^ ^172^ ^]^ fabricated an ACE2‐modified OFET to detect SARS‐CoV‐2 in saliva (Figure 9i). In this work, ACE2 was spin‐coated onto the pentacene semiconductor channel surface, diffused through the grain boundaries of the dendritic structure into the conducting channel, and coating at 5000 rpm balanced the device performance and sensitivity. A prototype module (SenFlex, Figure 9j) was designed for the detection of saliva samples diluted in DI water in 1:4 ratio, with a sample consumption of only 1 μL. As shown in Figure 9k, the sensor achieved detection in less than 1 min, with a high sensitivity of approximately 94%, and specific sample preparation or viral transfer medium was not required. Saliva testing is a rapid, painless, and noninvasive method that can be used as an alternative to nasopharyngeal swabs for mass screening and diagnosis.^[^ ^202^ ^]^ However, the resistance to interference demonstrated by biosensors in response to complex samples is a concern. The low specificity of clinical samples may limit its application in large‐scale screening.

### 5.2 Detection of SARS‐CoV‐2 Antibody

Following exposure to foreign antigens, the human body produces three main types of antibodies: IgA, IgG, and IgM. Of these, IgA and IgG levels persist, reflecting long‐term immune responses.^[^ ^203^ ^]^ Specific IgA and IgM antibodies against SARS‐CoV‐2 can be detected in clinical settings 7*d* after viral infection or 3–4 d after symptom onset. Specific IgG antibodies against the virus can be detected 7–10 d after SARS‐CoV‐2 infection. IgG titers increase within 3 wks of symptom onset. IgG levels begin to decline after the 8th week but remain above the detection threshold. Antibody testing is often more reliable for asymptomatic infected individuals and for false‐negative nucleic acid infections.^[^ ^204^ ^]^

**Figure 10:**
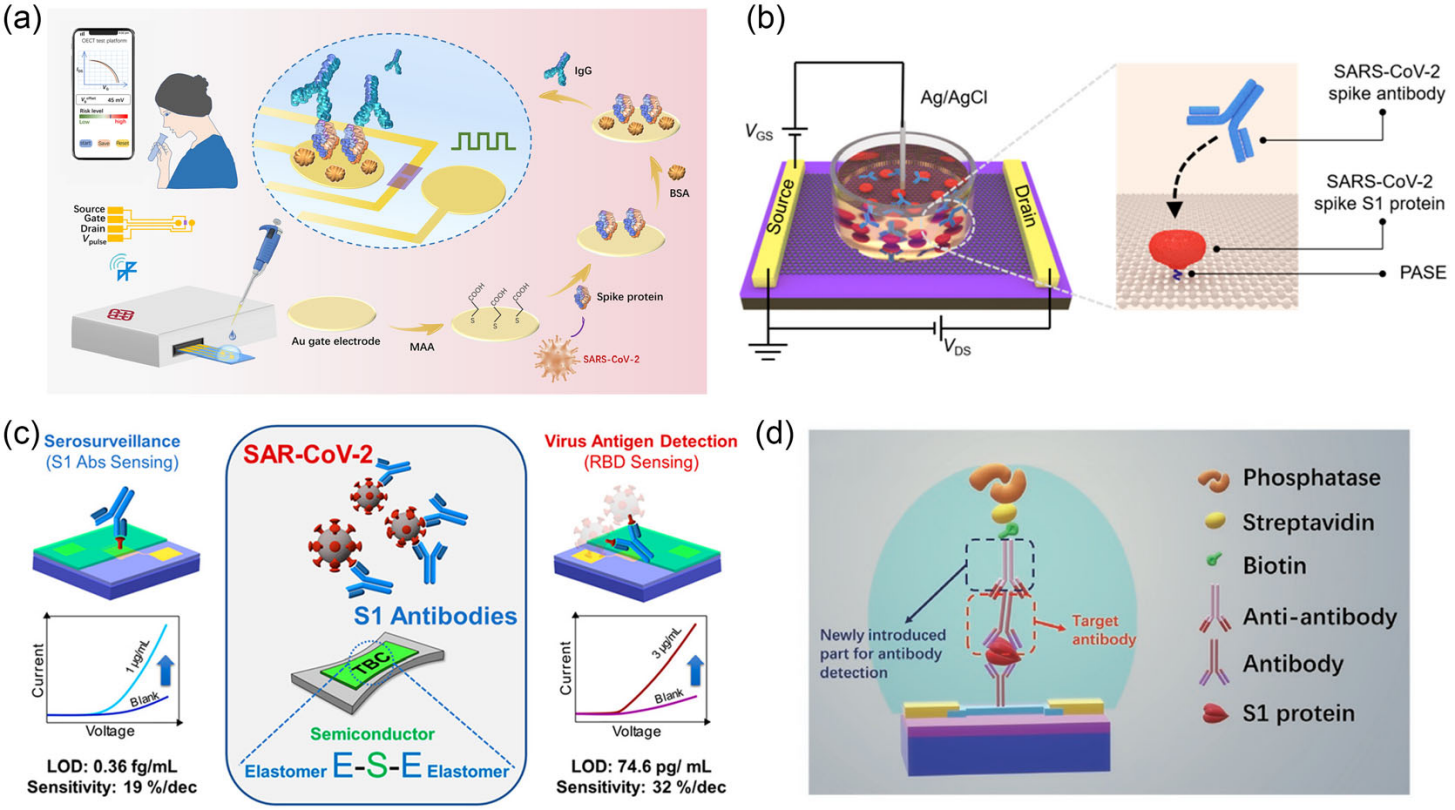
Transistor‐based biosensors for SARS‐CoV‐2 antibody detection. a) A schematic of the portable sensor and modification process of the gate electrode. Reproduced under the terms of the CC‐BY Creative Commons Attribution 4.0 International license (https://creativecommons.org/licenses/by/4.0).^[^ ^205^ ^]^ Copyright 2021, The Authors, published by the American Association for the Advancement of Science. b) Schematic of the g‐FET biosensor. Reproduced with permission.^[^ ^206^ ^]^ Copyright 2021, American Chemical Society. c) A schematic diagram of transistor detection where the active layer is TBC. Reproduced with permission.^[^ ^170^ ^]^ Copyright 2023, American Chemical Society. d) A schematic of the FET biosensor for detecting IgG. Reproduced with permission.^[^ ^209^ ^]^ Copyright 2022, Springer Nature.

Liu et al.^[^ ^205^ ^]^ developed an ultrafast, label‐free, and portable SARS‐CoV‐2 IgG detection platform based on OECTs controlled by mobile phones (**Figure** **10**a). The buffer concentration was first optimized by considering the effect of *λ*_D_ on the sensitivity of the device. The performance of the device improved at PBS buffer concentrations of 10 and 1 μm. The pH of the buffer was adjusted to enhance the sensitivity of the device, and a pulse voltage was applied to the gate electrode to improve the efficiency of antigen and antibody binding. The SARS‐CoV‐2 spike protein was immobilized on the gate electrode by covalent binding to the MAA. The positively charged IgG was bound to spike protein, changing the surface potential of the gate electrode and leading to a response from the sensor. The LOD of the sensor is 1 fm in PBS. Finally, the spike protein was diluted in serum and saliva for sensor performance testing (the serum samples were sterile‐filtered, and the saliva was diluted with 10 mm PBS to decrease its viscosity) with an LOD of 10 fm.

Kang et al.^[^ ^206^ ^]^ fabricated a GFET biosensor for detecting IgA in a full‐serum solution (Figure 10b). The spike S1 protein was immobilized on graphene to capture antibodies. The voltage generated by the drain‐source electrode attracts negatively charged antibodies, thus promoting effective biorecognition of antibodies and antigens. During this process, negatively charged antibodies act as electron donors for graphene, causing n‐type graphene doping. Therefore, a measurable electrical response was generated, and the LOD of the sensor was as low as 10^−18^ m. In addition, human serum served as the buffer, and clinical serum samples were measured to evaluate the practical application capability of the device. The test results of the sensor matched the qRT‐PCR test results perfectly, and the diagnostic sensitivity of the sensor was approximately 100%. The average diagnostic time was 5.3 min, and the shortest was 2 min.

MoS_2_ is a 2D semiconductor material, similar to graphene, with high carrier mobility and a large specific surface area.^[^ ^207^ ^]^ Wei et al.^[^ ^208^ ^]^ fabricated a MoS_2_ functionalized FET for detecting IgG in the sera of vaccinated test subjects. The MoS_2_ FETs were immersed in APTES, and then a GA solution was added to their surface for SARS‐CoV‐2 spike protein immobilization. The effective recognition of the antibody by the spike protein probes increased the conductivity of the devices and generated large current response signals. A calibration strategy was established for the quantitative analysis to avoid the effects of signal variations between different devices. After calibration, the signal coefficient of variation between devices was 6%, indicating good reproducibility between the devices. Finally, serum samples from the unvaccinated and vaccinated subjects were selected for performance testing. All samples were diluted 1000 times with PBS before testing. The sensor responded well to samples with antibodies, showing good sensitivity and selectivity.

To improve the application of OFETs in wearable devices, it is necessary to adjust the elastic modulus of the active layer to suit the skin stretchability. Ditte et al.^[^ ^170^ ^]^ synthesized a TBC as the active layer of OFET consisting of PDPP‐TT (a polymer‐conjugated semiconductor material) flanked by PDMS (Figure 10c). The TBC possesses a low modulus of 24 MPa, which ensures that there is no formation crack even at 90% strain. The TBC was modified to the OFET substrate by spin‐coating, and then the OFET was placed into a solution containing the SARS‐CoV‐2 Spike RBD protein. Modification of the antigen process was accomplished by the physical adsorption of PDMS. Therefore, PDMA provides not only tensile properties but also reliable physical adsorption of biomolecules. With the addition of IgG, the current signal of the sensor is enhanced, and the LOD of the sensor can reach 0.36 fg mL^−1^, whereas the sensitivity of the sensor is 19% decade^−1^. Block copolymer offer solutions for implementing wearable sensor platforms.

Chen et al.^[^ ^209^ ^]^ developed an FET with an In_2_O_3_ channel and a stable enzyme reporter to detect IgG (Figure 10d). IgG bound to In_2_O_3_ via 3‐phosphonopropioninc acid and specifically targeted the SARS‐CoV‐2 spike protein. After the captured components of the antigen and antibody were formed, IgG was added again for incubation. A biotin‐conjugated anti‐IgG antibody was used as a secondary antibody. Streptavidin‐conjugated phosphatases were introduced. When the enzymatic reaction begins, the deprotonated hydroxyl group on the surface of the In_2_O_3_ nanoribbon is protonated as the pH decreases and is converted into an electrical signal by transistor detection to complete the detection of IgG. The sensor exhibited a good detection limit for both PBS and human whole blood (1 pg mL^−1^).

### 5.3 Detection of SARS‐CoV‐2 Nucleic Acid

Antibodies are generally produced by humoral immune responses one week or more after viral infection.^[^ ^210^ ^]^ RNA is an important molecular biomarker of nucleic acids that appear earlier than antibodies. Therefore, nucleic acid testing has become an effective method for early diagnosis of viral infections.

Gao et al.^[^ ^149^ ^]^ fabricated a poly‐l‐lysine (PLL) modified GFET to detect SARS‐CoV‐2 RNA in throat swab samples. PLL is immobilized on graphene surfaces via electrostatic forces because of its rich cationic nature, and it can be combined with anionic DNA probes.^[^ ^211^ ^]^ In addition, PLL‐functionalized graphene does not affect the *λ*_D_ of the sensor, ensuring that the sensing performance is solely dependent on the immobilization density of the probe via the PLL. Different concentrations of SARS‐CoV‐2 RNA were added to throat swab solutions for simulated testing. Upon recognition of the probe and RNA, n‐doping of graphene occurs, causing an electrostatic charge in the graphene channel and a shift in the Dirac point to the left. Although the sensitivity of the sensor fluctuates among the various batches, the average sensitivity can reach up to 9 mV per decade. To further validate the accuracy of the gFET in actual sample detection, the SARS‐CoV‐2 pseudovirus was added to throat swabs for testing, and the detection accuracy was consistent with that of qPCR, indicating that the sensor has potential for practical applications.

**Figure 11:**
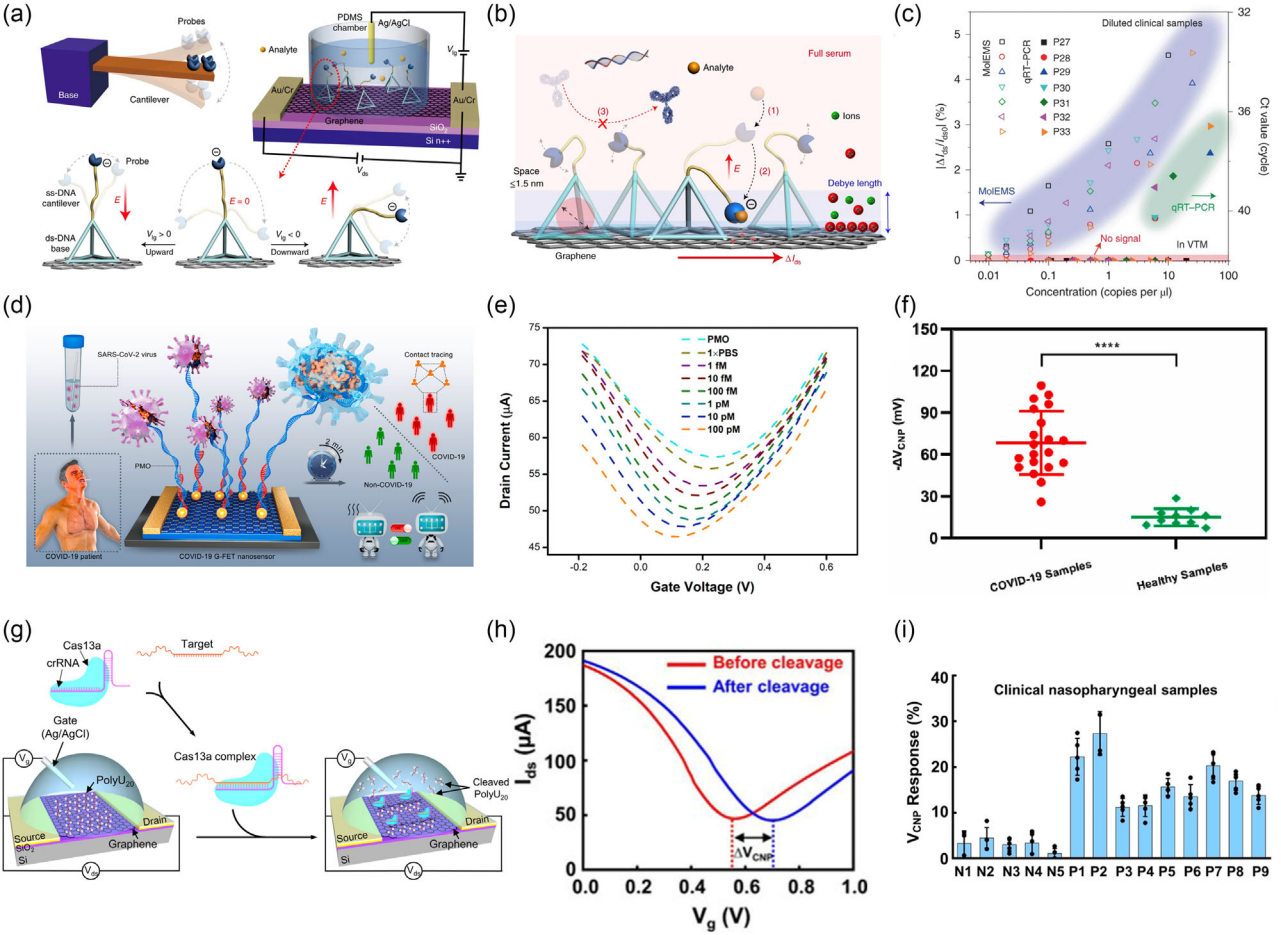
Transistor‐based biosensors for SARS‐CoV‐2 nucleic acid detection. a) A schematic of the MoIEMS and electrostatic driver. b) Sensing mechanism of MoIEMS. c) Response of the sensor to clinical samples and Ct values in VTM. a–c) Reproduced with permission.^[^ ^213^ ^]^ Copyright 2022, Springer Nature. d) A schematic of the PMO‐functionalized G‐FET sensor for detected SARS‐CoV‐2 RdRp RNA. e) The transfer curves of the device at the different concentrations of SARS‐CoV‐2 RdRp RNA. f) Significant differences between positive and negative samples. d–f) Reproduced with permission.^[^ ^216^ ^]^ Copyright 2021, Elsevier. g) A Schematic of the device and principles for unamplification detecting the SARS‐CoV‐2 RNA. h) Changes in the transfer curve of gFET after adding the target sequence. i) Signal changes of negative and positive in clinical samples. g–i) Reproduced with permission.^[^ ^229^ ^]^ Copyright 2022, Wiley‐VCH.

Microelectromechanical systems (MEMS) are highly integrated devices that convert biological, chemical, and other physical signals into electrical signals.^[^ ^212^ ^]^ By integrating with FET, MEMS can enable the fabrication of ultrasensitive biosensors. Wang et al.^[^ ^213^ ^]^ constructed a molecular electromechanical system (MolEMS) and a portable prototype device to detect SARS‐CoV‐2 RNA in unamplified samples. MolEMS‐integrated MEMS with FET. The MEMS consists of a tetrahedral ds‐DNA rigid base and a flexible ss‐DNA cantilever probe that can free to “surf” in solution and is modified on the graphene channel via PASE (**Figure** **11**a). The intensity of a signal in a high ionic strength buffer is often influenced by *λ*_D_, and effective recognition should occur within *λ*_D_. MolEMS addresses this limitation: when a negative *V*_Ig_ is applied, the resulting local electric field drives the negatively charged ssDNA cantilevers downward (Figure 11b). This causes the negatively charged SARS‐CoV‐2 RNA, after identification with the probe, to approach the graphene, leading to a change in the graphene channel potential. Thus, a change in the conductivity of the transistor completes the electrical signal output. Finally, nasopharyngeal swab samples were tested, and all samples were heat‐treated to release nucleic acids prior to testing. The designed system detects clinical samples with a low concentration down to approximately 0.02 copies μL^−1^ RNA in viral transport medium (VTM) and can be completed the test in approximately 0.1–4 min, which is faster than qRT‐PCR detection (Figure 11c).

Phosphorodiamidate morpholino oligomers (PMOs) are short ss‐DNA analogs with a neutral charge^[^ ^214^ ^]^ that possess good stability, high solubility, and specificity as ideal probe molecules for nanochannel sensing.^[^ ^215^ ^]^ Li et al.^[^ ^216^ ^]^ fabricated a PMO‐modified G‐FET to detect the SARS‐CoV‐2 RdRp RNA sequence (Figure 11d). AuNPs were deposited on RGO and modified with a PMO probe. After adding different concentrations of RNA and hybridizing with PMO, the negative charge of RNA led to the n‐doping of graphene and caused the transfer characteristic curve to shift to the left (Figure 11e). The LOD of the sensor was 0.37 fm in PBS. To verify the effectiveness of the PMO probe, the same base sequence and concentration of the ssDNA probe were used to replace PMO to detect SARS‐CoV‐2 RdRp RNA sequences. The data show that the signal response was much smaller than that of the immobilized PMO G‐FET, and there were statistically significant differences (*p* < 0.01) in the signal response between the two. Moreover, in the specificity test, the ssDNA‐modified G‐FETs showed a greater false signal for SARS‐CoV RdRp and one‐base‐mismatched SARS‐CoV‐2 RdRp RNA detection. The LOD of the sensor was 2.29 fm in throat swab and 3.99 fm in serum. The sensor completed the detection within 2 min, accurately distinguishing between healthy and infected individuals (Figure 11f), and the results were highly consistent with the RT‐PCR results.

Reverse transcriptase LAMP (RT‐LAMP) techniques have been considered alternative diagnostic tests to RT‐PCR because of their simplicity and shorter testing times.^[^ ^217^ ^]^ Park et al.^[^ ^218^ ^]^ fabricated a biosensor that combined the amplification capabilities of RT‐LAMP with a crumpled graphene FET (cgFET) to detect SARS‐CoV‐2 virus. Crumpled graphene is a 3D material, the surface of the convex region increases the *λ*_D_, which makes graphene more susceptible to electrical charges in the *λ*_D_, improving the sensitivity of the sensor.^[^ ^219^ ^]^ The amplified products were then diluted and transferred to a cgFET for testing. As the negative samples did not contain the viral genome, the ssDNA primers were not consumed during RT‐LAMP, resulting in a large amount of negatively charged ssDNA primers in the amplification product. The ssDNA primers were strongly absorbed on the surface of cgFET through noncovalent *π*–*π* stacking interactions, causing the n‐doping of graphene and the Dirac point shift. The signal response of the cgFET was significantly larger than that of the flat graphene sensor, demonstrating its high sensitivity. In addition, the inactivated SARS‐CoV‐2 virus spikes were detected in the VTM solution, the sensitivity and specificity of the sensor reached 100%, and reducing the test time to 1 h, including 30 min of amplification.

InSe is an emerging 2D semiconducting material that possesses high mobility owing to its small effective mass of carriers^[^ ^220^ ^]^ making it susceptible to Coulomb scattering and providing the possibility of the sensitive detection of biomolecules. Ji et al.^[^ ^221^ ^]^ fabricated a novel InSe‐FET biosensor to detect RNA segments in SARS‐CoV‐2 ORF‐1ab. The biosensor system was built on a silicon wafer to avoid the effect of the electric gating field induced by the liquid gate electrode on the interaction of the charged molecules. The sensor was then integrated with a microfluidic chip. DNA probes were injected through the microfluidic channels and immobilized on InSe via van der Waals forces. The negatively charged probes form Coulomb scattering centers on InSe, which produces the P‐doping of InSe. When the probes were saturated, they caused a drop in the FET‐to‐drain current. When throat‐swab RNA was injected and hybridized with DNA, the hybridization mixture was released from the InSe and flowed out of the microfluidic channel. This decreases the number of Coulomb scattering centers and the P‐doping effect, causing an increase in the FET‐to‐drain current. The sensitivity of the InSe‐FET biosensor was found to be 11.7461 decade^−1^, and it accurately identified one base mismatch of RNA. In addition, five independent sensors were used to test the same concentration of target RNA, and the output response had a RSD of 6.67%, indicating excellent reliability of the InSe‐FET biosensor.

Structural DNA nanotechnology can improve the binding of probes to targets through the design of different DNA configurations. Wu et al.^[^ ^222^ ^]^ fabricated a triple‐probe tetrahedral DNA framework (TDF) modified gFET to detect SARS‐CoV‐2 RNA. The top tetrahedron of the TDF was anchored to three DNA probes specifically recognized by RNA. Compared with the ssDNA structure, the TDF structure can effectively avoid the entanglement of nucleic acid chains on graphene, thus improving the sensitivity of detection. The three target SARS‐CoV‐2 RNAs are located in the ORF1ab, RdRp, and E gene regions. When the target RNA and probe hybridize, they induce charge accumulation on the graphene surface, causing doping of the graphene and signal transduction. Owing to the synergistic effect of the triple probes and the special nanostructure, a higher binding affinity, faster response, and better specificity were obtained. The sensor was able to detect 0.025–0.05 copy μL^−1^ of RNA in artificial saliva without amplification, which was lower than that of RT‐PCR. Furthermore, of the 30 nasopharyngeal swabs, all 14 positive cases were identified within a mean diagnostic time of 74 s. In the detection of nasopharyngeal swab samples, the sensor can achieve a sensitivity of 0.93 and specificity of 1, and the results are highly consistent with RT‐PCR. Finally, the 10‐in‐1 positive (negative) pooled samples were tested. The results revealed that the sensor could significantly distinguish 10‐in‐1 positive pooled samples from 10‐in‐1 negative pooled samples.

Owing to the high specificity and programmability of the clustered regularly interspaced short palindromic repeats (CRISPR)/CRISPR‐associated nuclease (Cas) systems, it has been rapidly investigated for use as a biological recognition element in nucleic acid biosensor systems. Subsequently, a variety of Cas proteins has been discovered and applied, such as Cas12 and Cas13, which have shown the ability to cut dsDNA and RNA with RNA‐guided target recognition, respectively^[^ ^223^, ^224^ ^]^ and efficiently cut nearby nontarget sequences.^[^ ^225^, ^226^ ^]^ Multiple CRISPR‐Cas systems have been used for highly sensitive and specific SARS‐CoV‐2 nucleic acid detection, such as SHERLOCK^[^ ^227^ ^]^ and DETECTR^[^ ^228^ ^]^ methods. However, additional fluorescent markers and multistep nucleic acid amplification processes need to be developed.

Li et al.^[^ ^229^ ^]^ fabricated a CRISPR‐Cas13a‐mediated GFET for the unamplified detection of SARS‐CoV‐2 RNA (Figure 11g). The device consisted of six CRISPR‐Cas13a‐GFETs devices on silicon wafers. A negatively charged RNA reporter (PolyU_20_) was immobilized on the graphene surface using PBASE. With the hybridization of RNA and the target nucleic acid, the trans‐cleavage activity of the CRISPR Cas13a endonuclease is triggered, leading to the separation of PolyU20 from the graphene channel. This reaction enhances the reduced electron transfer from the RNA phosphate backbones to the graphene channel, causing P‐doping of the graphene channel and a right shift of the Dirac point (Figure 11h). Owing to the advantages of the FET and the transactivity of the Cas13a endonuclease, the LOD of the sensor can reach 1 am. Finally, the sensor was able to detect heat‐inactivated and clinical SARS‐CoV‐2 nasal swab samples (the samples were heated at 95 °C for 5 min, along with Quick Extract solution for lysis viruses). The LOD reached 1 am, and its diagnostic performance was comparable to that of RT‐qPCR (Figure 11i). Recently, Yu et al.^[^ ^230^ ^]^ used the gene‐targeting ability of CRISPR‐Cas13a to construct a novel SGGT that allowed unamplified and label‐free SARS‐CoV‐2N gene RNA detection, showing a low LOD, reaching aM levels in both buffer and serum. Moreover, the detection results of the developed sensor were consistent with those of RT‐PCR when testing throat swab samples. Despite the discovery of the CRISPR‐Cas system and the development of related technologies that have inspired numerous advances in nucleic acid detection, off‐target issues remain worrisome. The catalytic site of Cas13a for RNA‐guided target cleavage is located on the external surface,^[^ ^231^ ^]^ which allows for the promiscuous cleavage of RNAs.^[^ ^232^ ^]^

## 6 Challenges and Perspectives

The recent COVID‐19 pandemic has underscored the urgent need for the rapid and accurate diagnosis of infectious diseases such as SARS‐CoV‐2. The engineering of transistor‐based biosensors has played a pivotal role in developing highly sensitive and specific biosensors for the detection of SARS‐CoV‐2, enabling the early diagnosis and treatment of COVID‐19, as summarized in **Table** **1**. Microfabrication techniques, including microelectromechanical system technology, are critical for achieving miniaturization and integration of biosensing systems, enabling precise control of device dimensions and spacing and the integration of various components into a single device.^[^ ^233^ ^]^ Functionalization of the transistor surface with specific bioreceptors and optimization of device parameters enable the specific recognition of SARS‐CoV‐2.

**Table 1:** Summary of transistor‐based biosensors for detection of SARS‐CoV‐2

Analyte type	Transducera)	Bio‐receptorsa)	Detection methoda)	Sample	LOD	Linear range	Detection time	Sensitivity	References
Spike protein	GFET	Antibody	Antibody/PBASE/Graphene	Nasopharyngeal swabs	2.42 × 10^2 ^copies mL^−1^	1.6 × 10^1^–1.6 × 10^4 ^pfu mL	–	–	[40]
	WSe_2_‐FET	Antibody	Antibody/MUA/WSe_2_	–	25 fg μL^−1^	25–10 ng μL^−1^	–	–	[121]
	GO/Gr FET	Antibody	Antibody/GO	Throat swab	8 fg mL^−1^	0.01–100 pg mL^−1^	20 min	11.68 mV decade^−1^	[192]
	Sc‐SWCNT FET	Antibody	Antibody/Sc‐SWCNT	Nasopharyngeal swabs	0.55 fg mL^−1^	5.5 fg mL^−1^–5.5 pg mL^−1^	–	Δ*I/I*_0_ = 25%	[198]
	GO‐FET	Antibody	Antibody/PBASE/Pt,Pd nanoparticles/GO	–	1 fg mL^−1^ in PBS	1 fg mL^−1^–100 ng mL	–	–	[240]
	Graphene based FET	ACE2	ACE2/PBASE/Graphene	Nasopharyngeal swabs	65 cps mL	2 μg mL^−1^–2 pg mL^−1^	–	–	[191]
	OFET	ACE2	ACE2/Pentacene	Saliva	–	–	≈20 s	Δ*I/I*_0_ = 94%	[172]
	GFET	ACE2	ACE2/PBASE/Graphene	–	2.94 am in PBS	–	–	–	[194]
	Poly‐si NWFET	Antibody	Antibody/GA/Nanowire	–	0.51 ag mL^−1^	250 ag mL^−1^–2.5 pg mL^−1^	–	0.19 decade^−1^	[241]
	HJ‐TFT	Antibody	Antibody/GA/APTES/ZnO	Serum	865 × 10^−18 ^ m in 0.1 × PBS	10 am–100 pm	<2 min	–	[92]
	CNT‐FET	Antibody	Antibody/PBASE/CNT	Simulated saliva	4.12 fg mL^−1^	0.1 fg mL^−1^–5.0 pg mL^−1^	2–3 min	Δ*I/I*_0_ = 91.18%	[242]
	Dual‐gate TFT	Antibody	Antibody/APTES/ITO	Cultured virus	1.17 fg mL^−1^	1 fg mL^−1^–1 ng mL	–	Δ*I/I*_0_ = 163 ± 13.6% to 1 pg mL^−1^	[243]
	OECT	Antibody	Antibody/Nanobody/linker/SpyCatcher/SpyTag /HDT/Au	Nasopharyngeal swab	1.2 × 10^−21 ^ m in saliva	10^−20^–10^−8 ^M	10 min	–	[74]
	MXene GFET	Antibody	Antibody/APTES/Mxene‐graphene	–	1 fg mL^−1^ in PBS	1 fg mL^−1^–10 pg mL^−1^	50 ms	–	[244]
	LIG FET	Antibody	Antibody/Graphene	Serum	1 pg mL^−1^ in PBS	1 pg mL^−1^–1 μg mL^−1^	15 min	0.2 V decade^−1^	[9]
Antibody	OECT	S protein	Spike protein/MAA/Au gate	Serum and saliva	1 fm in PBS	10 fm–100 nm in serum	5 min	–	[205]
	GFET	S protein	Spike protein/PASE/Graphene	Serum	2.6 × 10^−18^ m in PBS	5.0 × 10^−18^–5.0 × 10^−12 ^M	5.3 min	–	[206]
	MoS_2_ FET	S protein	S protein/APTES/GA/MoS_2_	Serum	–	–	–	–	[208]
	OFET	S protein	Spike protein/TBC	–	0.36 fg mL^−1^ in PBS	0.1 fg mL^−1^–1 μg mL^−1^	–	Δ*I/I*_0_ = 19% decade^−1^	[170]
	FET	S protein	Phosphatase/Streptavidin/Biotin/Antibody/Protein/In_2_O_3_	whole blood	1 pg mL^−1^ in PBS and blood	1 ng mL^−1^–1 pg mL^−1^	–	–	[209]
Nucleic acid	Graphene FET	ssDNA	ssDNA/PLL/Graphene	Throat swab	–	1 fm–100 pm	–	9 mV decade^−1^	[149]
	Graphene FET	ssDNA	ssDNA/MEMS /Graphene	Nasopharyngeal swab	≈0.02 copies μL^−1^	1.67 × 10^−19^–8.33 × 10^−17^ M	4 min	Δ*I/I*_0_ = 0.8 to 6.85%.	[213]
	Graphene FET	PMO	PMO/AuNPs/RGO	Throat swabs and serum	0.37 fm in PBS	1 fm–100 pm in 0.01 × PBS	2 min	–	[216]
	Crumpled GFET	ssDNA primers	Graphene	Inactivated virus	–	–	1 h	–	[218]
	FET	ssDNA	ssDNA/InSe	Throat swab	–	–	–	11.7461 decade^−1^	[221]
	GFET	TDF dimer	TDF dimer/PASE/Graphene	Nasopharyngeal swabs	0.01 copy μL^−1^	0.025–0.05 copy μL^−1^ in artificial saliva	74 s	Δ*I/I*_0_ = 93%	[222]
	GFET	CRISPR‐Cas13a	PolyU_20_/Graphene	Nasal swab	1 am	1 am–1 pm	30 min	–	[229]
	SGGT	CRISPR‐Cas13a	Cas13a‐RNPs/SAM‐dextran/Au	Throat swab	1.3 × 10^−17 ^ m in PBS	10^−17^–10^−11^ m in PBS	10 min	–	[230]
	Extended‐gate FET	Ion‐sensitive	–	Wastewater	0.31 × 10^−13^ ng μL^−1^	0.01–10 ng μL^−1^	15 min	56.2 μA decade^−1^	[178]
	FET	ssDNA	ssDNA/AuNPs/Al_2_O_3_	–	0.79 fm for RNA	10^−14^–10^−8^ M	15 min	–	[245]

a) Abbreviations: FET: field‐effect transistor. PBASE: 1‐pyrenebutyric acid *N*‐hydroxysuccinimide ester. PBS: phosphate‐buffered saline. MUA:11‐mercaptoundecanoic acid. GO/Gr: graphene oxide/graphene. Sc‐SWCNTs: semiconducting single‐walled carbon nanotube. HJ: heterojunction. TFT: thin‐film transistor. NWFET: nanowire field‐effect transistor. APTES:3‐aminopropyltriethoxysilane. CNT: carbon nanotube. AA: ammonium acetate. ITO: indium tin oxide. HDT:1,6‐hexanedithiol. UTM: universal transport medium. LIG: laser‐induced graphene. GA: glutaraldehyde. TBC: triblock copolymer. MEMS: microelectromechanical systems. TDF: tetrahedral DNA framework. SGGT solution‐gated graphene transistor. CRISPR‐Cas: clustered regularly interspaced short palindromic repeats (CRISPR)/CRISPR‐associated nuclease. RNPs: ribonucleoprotein complexes. SAM: self‐assembled monolayer. AuNPs: gold nanoparticles.

**Figure 12:**
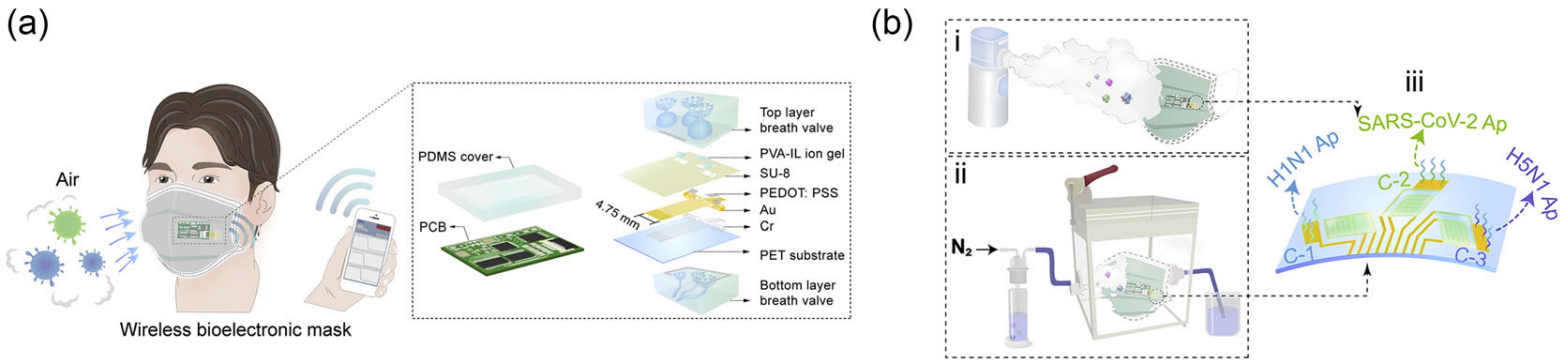
A wearable bioelectronic mask for detecting SARS‐CoV‐2. a) Diagram of a bioelectronic mask. b) PIL‐IGT detection schematic and multichannel modification schematic. a,b) Reproduced with permission.^[^ ^36^ ^]^ Copyright 2022, Elsevier.

Portable devices integrate sensors and digital circuitry to provide controllable and timely feedback for the detection of biomarkers in biofluids. Therefore, it is a potential tool for the detection of changes in biological signals.^[^ ^234^ ^]^ Wang et al.^[^ ^36^ ^]^ developed a wearable bioelectronic mask for detecting infectious respiratory viruses by integrating a multichannel ion‐gated transistor and IoT technology, as shown in (**Figure** **12**a). An ion‐gated transistor (PIL‐IGT) uses PET as the substrate, which allows for the bending function of the mask. An ionic gel (PVA‐IL) was prepared using PVA as the dielectric layer and PDOT: PSS as the channel material. The prepared PVA‐IL ionic gel exhibited recoverable elasticity at over 400% strain and maintained stable electrical performance, meeting the flexibility requirements of wearable devices. As shown in Figure 12b, the aptamer was immobilized on the gold gate electrode, and conformational rearrangement of the aptamer occurred when it bound to the SARS‐CoV‐2 spike protein. With an increase in the concentration of the spike protein, the surface charge concentration and distribution of the gate electrode change, altering the effective gate voltage on the gate electrode and generating a detectable electrical signal. The bioelectronic mask was connected to a wireless network via an internal microcontroller with a remote communication microchip, allowing for real‐time in situ detection of air. The system rapidly detects a signal response within 10 min, and LOD reaches 0.1 fg mL^−1^ when exposed to a viral atomizing gas and carrier gas. Additionally, different aptamers can be immobilized on the three channels of the transistor to achieve multichannel detection. In conclusion, wearable masks offer a new preselection method for the portable detection of respiratory viruses. However, other types of virus testing often require sample preprocessing. Achieving sample processing and multiplexing capabilities is an urgent problem for the realization of wearable devices.

For viral detection, consider the diversity and complexity of actual samples is also necessary. Microfluidics is a promising technology that uses channels of tens to hundreds of micrometers to process or manipulate small amounts of liquids.^[^ ^235^ ^]^ Its small size makes it a preselected tool for wearable devices. Najjar et al.^[^ ^236^ ^]^ established a LOC platform for the detection of SARS‐CoV‐2. The platform includes a sample preprocessing module and an electrochemical sensor module, enabling the integration of sample pretreatment and detection. The detection of three viral antigens is achieved by modifying three antibodies on the sensor electrode using the CRISPR system in trans cleavage, and the specificity and sensitivity of detection could reach 100%. Although the platform requires a small peristaltic pimp advancement reaction progress of the sample on the chip, the strategy of integrating sample preprocessing and designing different probes on the receptor surface for multichannel detection is sufficient to provide inspiration. Digital microfluidics (DMF) is a new method of automating sample analysis. The platform achieves automated addressing of analytes by controlling the wettability of droplet‐like analytes, which completes analyte preprocessing, detection, and signal output.^[^ ^237^ ^]^ Sun et al.^[^ ^238^ ^]^ established a platform in which DMF was combined with recombinase polymerase amplification (RPA)‐Cas12a‐based assay to detect SARS‐CoV‐2. Computer‐controlled movement of nucleic acids completes the processes of nucleic acid amplification and probe identification. Using the fluorescence signal change caused by the Cas12a enzyme cleavage reporter probe for complete detection, a built‐in camera function microscope was used to collect the signals. The detection time for the entire system was less than 30 min. The entire process was completed inside the chip, thereby reducing aerosol contamination. For the detection of clinical samples, the results were consistent with those of qRCP. The dynamically configurable nature of DMF offers the advantage of integration into wearable devices; however, most DMF devices still require an external power supply and complex circuit design, which limits further portability. Future combinations of portable wearables with microfluidic networks will provide a more convenient, fast, and intelligent model for multichannel analysis. Microfluidic low‐volume assays offer a broad range of applications in portable and wearable devices. In addition, the size of the devices can be reduced by building 3D structures.^[^ ^239^ ^]^ With smart big data technology, big data collection and storage, portable and wearable microfluidic platforms are expected to eliminate the requirement for professionalism and be widely used.

A multidisciplinary approach is required to develop advanced biosensing platforms that integrate expertise in materials science, electrical engineering, biochemistry, and microfabrication. This approach facilitates the standardized preparation of biosensors, with the potential to revolutionize the fields of diagnostics and personalized medicine. Biosensors enable the rapid and accurate diagnosis of diseases, monitoring treatment efficacy, and detection of environmental contaminants. The continued development of transistor‐based biosensors holds significant potential for advancing the field of biosensing and improving human health, especially in the context of infectious diseases, such as COVID‐19.

## 7 Conclusion

FETs have attracted significant attention as a promising detection technology for biosensing applications. This review provides a comprehensive overview of the application of functionalized transistors in the label‐free detection of SARS‐CoV‐2, focusing on the device design, probe immobilization methods, and testing approaches for nucleic acids, antigens, and antibodies. The combination of electronic devices and biological interfaces through the functionalization of transistors shows the unique detection advantages of transistor‐based biosensor technology, with ease of operation, programmability, and modular capabilities, which are lacking in other sensors. Compared with clinical detection technologies, biosensors exhibit superior sensitivity. The development of FET biosensors has the potential to provide rapid and accurate detection of viruses, helping to mitigate their spread and impact on public health. This review provides valuable insights into the use of functionalized transistors for biosensing and highlights the potential of biosensors to revolutionize the field of biodetection.

## Conflict of Interest

The authors declare no conflict of interest.
